# Viral infection induces inflammatory signals that coordinate YAP regulation of dysplastic cells in lung alveoli

**DOI:** 10.1172/JCI176828

**Published:** 2024-10-01

**Authors:** Xiuyu Lin, Weicheng Chen, Guilin Yang, Jiazhu Zhang, Huilin Wang, Zeyu Liu, Ying Xi, Tao Ren, Bo Liu, Pengfei Sui

**Affiliations:** 1 State Key Laboratory of Multi-Cell Systems, Shanghai Institute of Biochemistry and Cell Biology, Center for Excellence in Molecular Cell Science, Chinese Academy of Sciences, Shanghai , University of Chinese Academy of Sciences, China.; 2 Cardiothoracic Surgery Department, Children’s Hospital of Fudan University, Shanghai, China.; 3 Key Laboratory of Immune Response and Immunotherapy, Shanghai Institute of Immunity and Infection, Chinese Academy of Sciences, Shanghai, China.; 4 Department of Respiratory Medicine, Shanghai Jiao Tong University Affiliated Sixth People’s Hospital, Shanghai, China.; 5 School of Life Science and Technology, ShanghaiTech University, Shanghai, China.

**Keywords:** Pulmonology, Adult stem cells, Influenza

## Abstract

Severe viral pneumonia can induce rapid expansion of KRT5^+^ basal-like cells in small airways and alveoli; this forms a scar-like structure that persists in the injured alveoli and impedes normal alveolar epithelium regeneration. In this study, we investigated the mechanism by which viral infection induced this remodeling response. Through comparing different lung-injury models, we demonstrated that infection induced strong IFN-γ signal–stimulated dysplastic KRT5^+^ cell formation. Inactivation of interferon receptor 1 (*Ifngr1*) reduced dysplastic cell formation, ameliorated lung fibrosis, and improved lung-function recovery. Mechanistically, IFN-γ regulated dysplastic cell formation via the focal adhesion kinase (FAK)/Yes-associated protein 1 (YAP) pathway. Inhibiting FAK/Src diminished IFN-γ–induced YAP nuclear translocation and dysplastic cell formation. Inhibiting YAP during viral infection prevented dysplastic cell formation, whereas inhibiting YAP in persistent KRT5^+^ cells led to their conversion into distal club cells. Importantly, human dysplastic cells exhibited elevated FAK and YAP activity, and IFN-γ treatment promoted the transformation of human alveolar progenitor cells into dysplastic cells. These findings uncover the role of infection-induced inflammatory response in alveolar remodeling and may provide potential therapeutic avenues for the treatment of alveolar remodeling in patients with severe viral pneumonia.

## Abstract 2

Infection induced inflammation signal promotes lung alveolar remodeling with dysplastic KRT5+ cell formation

## Introduction

Pathogen invasion and environmental insults are common causes of lung injury. The regeneration of injured lung epithelium is mediated by lung progenitor cells. Although several types of progenitor cells can contribute to lung regeneration, the activation and contribution of progenitor cell types vary depending on the nature and severity of the injury (1–4). Identifying the injury-induced signal that activates certain types of lung progenitor cells will provide insights into understanding how the activation of progenitor cell types and regenerative pathways is regulated in different lung-injury settings, especially the activation of the dysplastic remodeling pathway, which leads to chronic impact on lung function.

The gas-exchanging epithelium in the lung alveoli is composed of alveolar type 1 cells (AT1s), which are responsible for gas exchange, and alveolar type 2 cells (AT2s), which make surfactant protein to prevent alveoli collapse and function as progenitor cells to maintain alveolar epithelium integrity (3, 5). It has long been shown that AT2 cells, bronchoalveolar stem cells (BASCs), and airway club cells are facultative progenitor cells that can repair injured alveoli via generating AT1 and AT2 cells (1, 2, 4, 6). However, recent studies showed that following respiratory viral infections, such as influenza and Sendai virus, a group of rare distal airway progenitor cells are activated and repair the injured alveoli with KRT5^+^ dysplastic cells (7–10). Though the integrity of alveolar epithelial barrier is restored through this dysplastic repair pathway, these KRT5^+^ cells were thought pathological, as they barely differentiate into alveolar epithelial cells, but generate ectopic goblet cells and tuft cells (8, 11–13). In human patients with severe influenza or COVID-19 infection, extensive expansion of dysplastic KRT5^+^ cells correlates with long-term compromised lung function after viral clearance (14–16). Although ectopic expansion of dysplastic KRT5^+^ cells in the alveoli is frequently induced by severe respiratory viral infection, the mechanism that activates this dysplastic response and how these dysplastic cells maintain their identity within injured alveoli are still unclear.

We studied the potential mechanism that controls the development of dysplastic remodeling during viral infection. We identified an immune-epithelial module mediated by IFN-γ that promoted dysplastic cell expansion in viral-infected lungs. IFN-γ coordinated dysplastic cell expansion via the focal adhesion kinase (FAK)/Yes-associated protein 1 (YAP) signaling axis. We further demonstrated that YAP is required for both the expansion and maintenance of dysplastic cells, which provides a potential therapeutic target that not only prevents dysplastic cell expansion, but also reduces the persistent dysplastic cells in the alveoli.

## Results

### Robust expansion of dysplastic KRT5^+^ cells occurs following strong antiviral immune response.

**Figure 1:**
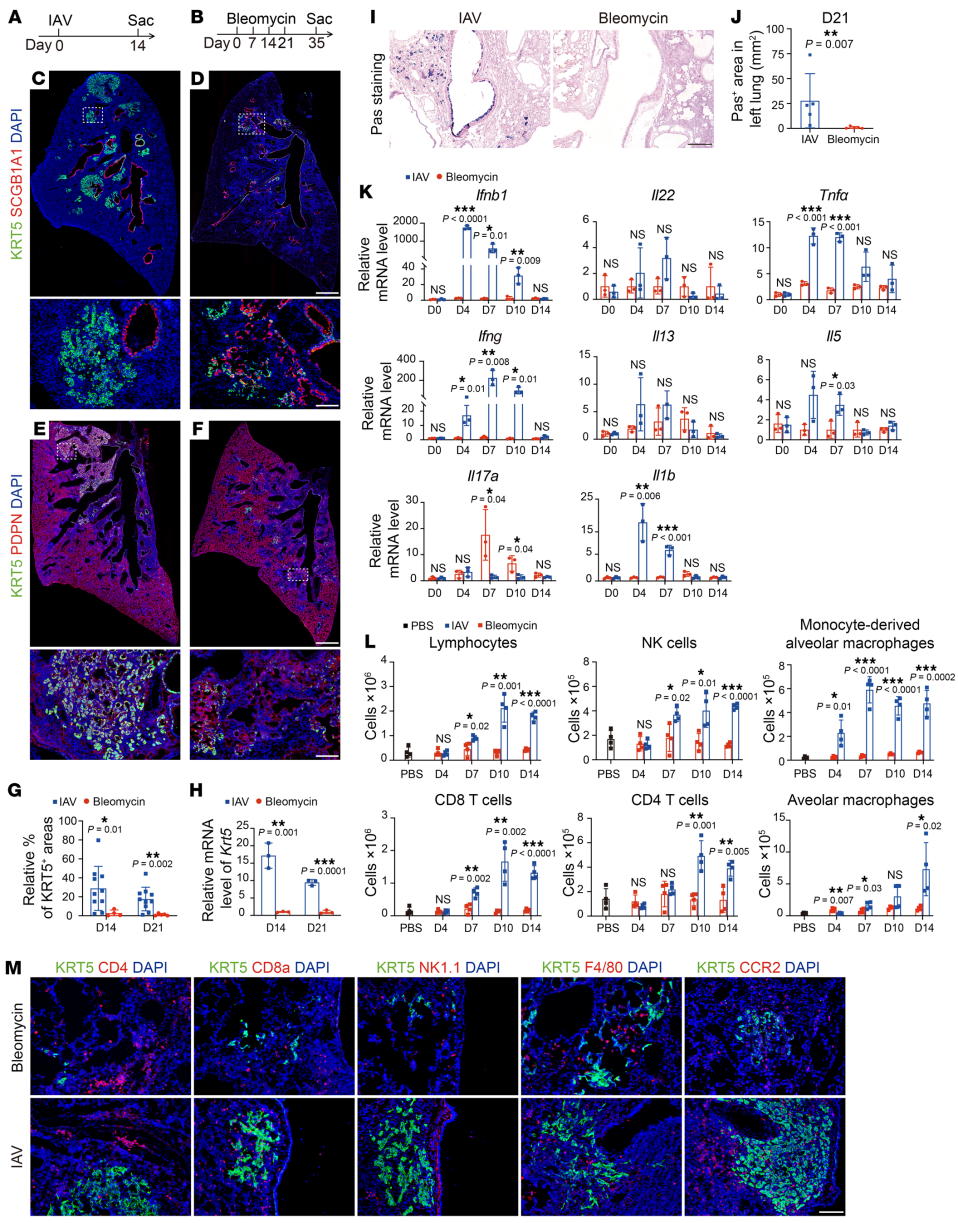
Comparison of lung histology and inflammatory response between IAV- and bleomycin-injured lungs. (**A** and **B**) Illustration of IAV- and bleomycin-induced lung injury models. Sac, sacrifice. (**C** and **D**) Immunofluorescence staining for KRT5 and SCGB1A1 in IAV- and bleomycin-injured lungs. Data are representative of sections from 3 mice. Scale bars: 500 μm (top row); 50 μm (bottom row). (**E** and **F**) Immunofluorescence images of dysplastic cells (KRT5^+^ PDPN^+^) and AT1 (PDPN^+^) cells. Scale bars: 500 μm (top row); 50 μm (bottom row). (**G**) Quantification of percentages of KRT5^+^ lung areas in total damaged alveolar areas (PDPN^–^ and KRT5^+^) in IAV- and bleomycin-injured lungs (*n* ≥ 4 mice per group). (**H**) *Krt5* expression was assayed by qRT-PCR in IAV- and bleomycin-injured lungs (*n* = 3 mice per group). (**I** and **J**) PAS staining and quantification of goblet cells in IAV- and bleomycin-injured lungs (*n* = 5 mice per group). Scale bar: 50 μm. (**K**) Inflammatory factor mRNA expression was assayed by qRT-PCR in IAV- and bleomycin-injured lungs at indicated time points (*n* = 3 mice per group). (**L**) Flow cytometry analysis of immune cells from PBS-, repetitive bleomycin–, or IAV-treated lungs at indicated time points (*n* = 4 mice per group). (**M**) Immunofluorescence staining for CD4, CD8a, F4/80, CCR2, or NK1.1 with KRT5 in IAV- and bleomycin-injured lungs at 14 dpi. Data are representative of sections from 3 mice. Scale bar: 50 μm. *P < 0.05; **P < 0.01; ***P < 0.001. Error bars represent means ± SEM. Multiple *t* tests (**H**, **K**, and **L**); 2-tailed Mann-Whitney *U* test (**G** and **J**).

To investigate the mechanism that regulates dysplastic KRT5^+^ cell expansion following influenza A virus (IAV) (H1N1 PR8 strain) infection, we first compared the lung histology in mice subjected to IAV to that of those challenged by bleomycin, a chemotherapy reagent that can also induce severe alveolar damage, but that has a limited extent of alveolar KRT5^+^ cell formation (8). We confirmed that both injury models led to comparable levels of lung histological change and similar levels of alveolar epithelial cell ablation (Supplemental Figure 1, A–D; supplemental material available online with this article; https://doi.org/10.1172/JCI176828DS1). Intriguingly, the dysplastic KRT5^+^ cells from IAV-infected lungs tend to form the typical KRT5^+^ “pod” structure, whereas most of the KRT5^+^ dysplastic cells in bleomycin-injured lungs formed a luminal-like structure and were frequently colocalized with SCGB1A1^+^ club cells (Figure 1, A–D). IAV-infected mice also showed much larger KRT5^+^ lung areas and higher *Krt5* mRNA levels compared with those challenged by bleomycin (Figure 1, E–H). Additionally, IAV-infected lungs showed goblet cell hyperplasia in both the airway and alveoli, whereas bleomycin-injured lungs exhibited virtually no goblet cells (Figure 1, I and J). Thus, these results suggest that additional factors in IAV-infected lungs stimulate KRT5^+^ cell expansion.

Inflammatory signals have recently been identified as drivers for tissue regeneration (17). We therefore compared the mRNA expression of immune factors in mice injured by these 2 models. We found that IAV-infected lungs showed much higher levels of *Ifnb1* and *Ifng* compared with those challenged by bleomycin (Figure 1K). Interestingly, the expression of *Ifng* reached its peak around day 7, when dysplastic KRT5^+^ cells first appeared in the distal airway, and remained at a high level until at least day 10, when dysplastic KRT5^+^ cells started to form large pod-like structures (18). In contrast, the expression of other cytokines we tested was either moderately induced, such as *Il17*, *Il22*, *Il13*, *Il5*, and *Tnfa*, or returned to baseline levels before the appearance of KRT5^+^ cells in the alveoli, such as *Il1b* (Figure 1K). These observations were also supported by the data from measuring inflammatory cytokine levels in bronchoalveolar lavage fluid (BALF) and lung homogenate (Supplemental Figure 1, E and F). Consistent with the high expression of inflammatory cytokines, IAV-infected lungs showed more infiltrated lymphocytes, NK cells, CD4^+^ T cells, monocyte-derived alveolar macrophages, and CD8^+^ T cells than bleomycin-injured lungs (Figure 1L and Supplemental Figure 1G). Costaining for markers of immune cells with dysplastic KRT5^+^ cells revealed that CD8^+^ T cells, the major source of IFN-γ during viral infection (19), were recruited to injured alveoli areas, including KRT5^+^ pods. Intriguingly, at day 28 after injury, we still observed abundant CD8^+^ T cells in the injured alveoli area (Supplemental Figure 1H). We also detected abundant CCR2^+^ monocyte–derived alveolar macrophages in the injured lung area, consistent with their role in tissue remodeling (20). However, NK cells, alveolar macrophages, and CD4^+^ T cells were less frequently detected in KRT5^+^ areas (Figure 1M). In contrast, very few of these immune cells were found in KRT5^+^ lung areas of bleomycin-injured lungs (Figure 1M). Thus, these observations suggest that the expansion of dysplastic KRT5^+^ cells in viral-infected lungs coincides with the robust antiviral inflammatory response, suggesting a role of inflammatory signals in controlling this process.

### Infection-induced IFN-γ is required for dysplastic remodeling in IAV-infected lungs.

**Figure 2:**
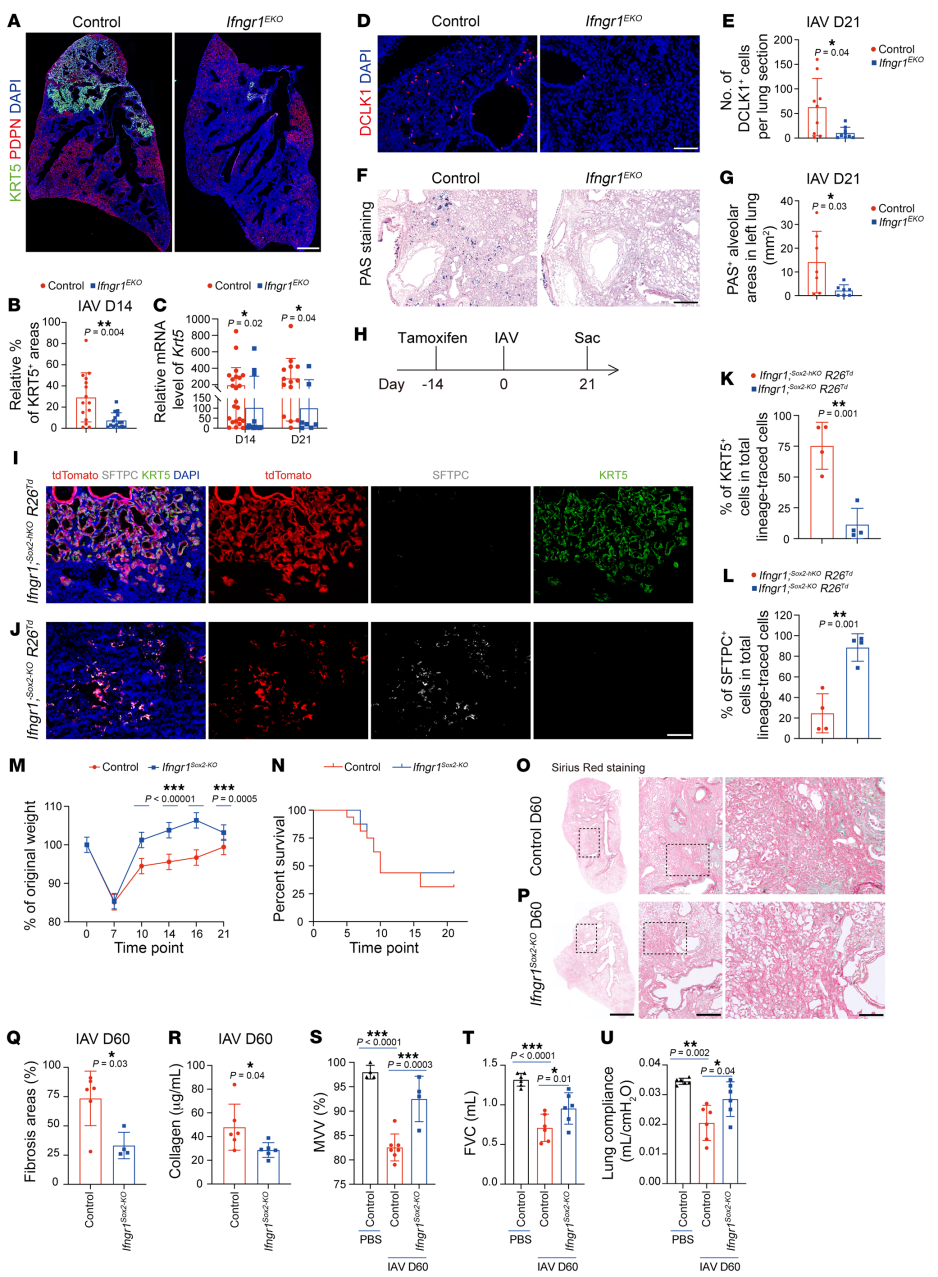
Epithelial IFN-γ signaling is essential for IAV-induced dysplastic KRT5^+^ cell formation. (**A** and **B**) Immunofluorescence analysis and quantification of percentages of KRT5^+^PDPN^+^ areas in PDPN^–^ and KRT5^+^ areas in control and *Ifngr1*^EKO^ mice at 14 dpi (*n* = 15 mice per group). Scale bar: 500 μm. (**C**) *Krt5* expression analysis in control and *Ifngr1*^EKO^ mice at indicated time points (*n* ≥ 7 mice per group). (**D** and **E**) DCLK1^+^ tuft cells detected in control and *Ifngr1*^EKO^ mice at 21 dpi (*n* = 9 mice per group). Scale bar: 50 μm. (**F** and **G**) Histological analysis of goblet cells in control and *Ifngr1*^EKO^ mice at 21 dpi (*n* = 7 mice per group). Scale bar: 50 μm. (**H**–**L**) Experiment design and immunofluorescence analysis and quantification of lineage-traced KRT5^+^ and SFTPC^+^AT2 cells in control and *Ifngr1*^Sox2–KO^ mice at 21 dpi (*n* = 4 mice per group). Scale bar: 50 μm. (**M** and **N**) Weight loss and survival curves of control and *Ifngr1*^Sox2–KO^ mice (*n* = 7 mice per group in **M**, *n* = 16 mice per group in **N**). (**O**–**Q**) Sirius red staining and quantification of fibrotic areas in control and *Ifngr1*^Sox2–KO^ mice at 60 dpi (*n* ≥ 4 mice per group). Scale bars: 500 μm (left row); 100 μm (middle row); 50 μm (right row). (**R**) Collagen content in control and *Ifngr1*^Sox2–KO^ mice at 60 dpi (*n* = 6 mice per group). (**S**–**U**) MVV, FVC, and lung compliance in indicated mouse groups at 60 dpi (*n* ≥ 4 mice per group). *P < 0.05; **P < 0.01; ***P < 0.001. Error bars represent means ± SEM. Two-tailed Mann-Whitney *U* test (**B**, **C**, **E**, **G**, **Q**, and **R**); 2-tailed Student’s *t* test (**K** and **L**); multiple *t* test (**M**); Kaplan-Meier test (**N**); 1-way ANOVA (**S** and **T**); Brown-Forsythe and Welch’s ANOVA (**U**).

Interferon signaling is critical to the host antiviral response and can directly signal to lung epithelial cells (19). Considering the potential link among infiltration of CD8^+^ T cells, expression kinetics of IFN-γ, and dysplastic KRT5^+^ cell formation after IAV infection, we hypothesized that IFN-γ may contribute to the formation of dysplastic KRT5^+^ cells. To test this, we inactivated interferon receptor 1 (*Ifngr1*) via *Shh^cre^* (hereafter *Ifngr1^EKO^*), in which Cre recombinase is expressed in all the lung epithelial cells (21). *Ifngr1^EKO^* mice showed normal lung epithelial cell composition and alveolar morphology compared with control mice, as indicated by immunofluorescent staining of epithelial cell-type–specific markers and histological staining (Supplemental Figure 2, A–E). Remarkably, following IAV infection, *Ifngr1^EKO^* mutant lungs showed a reduction in the KRT5^+^ lung area and decreased *Krt5* mRNA expression levels compared with control lungs (Figure 2, A–C). The numbers of alveolar DCLK1^+^ tuft cells and alveolar goblet cells, which are both derived from dysplastic KRT5^+^ cells, were also decreased (Figure 2, D–G) (12, 13, 22). In contrast, the goblet cell hyperplasia response in the airway occurred normally in *Ifngr1^EKO^* mutant lungs, suggesting that these cells are not derived from KRT5^+^ cells (Supplemental Figure 2, F and G). Further analysis revealed that the reduced formation of KRT5^+^ cells in *Ifngr1^EKO^* mice was not due to changes in viral clearance efficiency, as the *Ifngr1^EKO^* mutant mice and control mice showed comparable levels of viral nucleoprotein (NP) and nonstructural protein 1 (NS1) during lung recovery (Supplemental Figure 2H).

To determine whether *Ifngr1* is also required for the formation of dysplastic KRT5^+^ cells in bleomycin-injured lungs, we exposed control and *Ifngr1^EKO^* mutant mice to bleomycin challenge and found that the formation of dysplastic KRT5^+^ cells was not affected, indicating a dispensable role of IFN-γ signaling in the formation of dysplastic KRT5^+^ cells in bleomycin-injured lungs (Supplemental Figure 2, I–K). Thus, these data demonstrate that IFN-γ specifically regulates the formation of dysplastic cells induced by IAV, but not bleomycin.

### Inhibition of IFN-γ signaling promotes lung recovery following IAV infection.

In addition to intrapulmonary p63^+^ cells, the SOX2^+^ airway epithelial cells harbor multiple progenitor cell populations, such as club cells and BASCs, which can generate fresh AT2 cells to repair lung alveoli (10, 23). To determine whether inhibition of epithelial IFN-γ signaling affects lung regeneration, we generated *Sox2^creERT2^;Ifngr1^fl/fl^;R26-tdTomato* mice (hereafter *Ifngr1^Sox2–KO^*) to trace the fate of SOX2^+^ progenitor cells while inactivating *Ifngr1* (Figure 2H). Consistent with the phenotype of *Ifngr1^EKO^* mice, the expansion of KRT5^+^ dysplastic cells was largely suppressed in lungs from *Ifngr1^Sox2–KO^* mice after IAV infection (Figure 2, I and J). Notably, the mutant lungs showed an increase in lineage-traced AT2 cells in the injured alveoli (Figure 2, K and L). Although the weight loss at the early stage and animal survival were not different between control and mutant mice, the mutant exhibited improved body weight recovery after day 10 (Figure 2, M and N). Consistently, the mutant mice showed improved functional lung recovery, including maximal voluntary ventilation (MVV), forced vital capacity (FVC), and lung compliance, and ameliorated fibrosis at 2 months after infection (Figure 2, O–U). These findings demonstrate that disruption of epithelial IFN-γ signaling promotes lung recovery following IAV infection.

### IFN-γ regulates lung dysplastic remodeling in a Stat1-independent manner.

The canonical IFN-γ signaling acts through the JAK/STAT signaling axis to relay IFN-γ signals to the nucleus and activate downstream gene expression (24). To determine whether IFN-γ regulates dysplastic KRT5^+^ cell expansion through the JAK/STAT pathway, we used JAK1/JAK2 antagonist or *Shh^Cre^;Stat1^fl/fl^* (here after *Stat1^EKO^*) mutant mice to test the role of JAK/STAT signaling in this process. Using an established organoid culture system (10), we purified and cultured primary intrapulmonary p63^+^ progenitor enriched cells (EpCAM^+^Integrin β4^+^). Treatment of cultured intrapulmonary p63^+^ cells with IFN-γ promotes their transdifferentiation into KRT5^+^ cells, and this effect was diminished when baricitinib or fedratinib, specific antagonists for JAK1 and JAK2 (25), was added to the culture medium (Supplemental Figure 3, A and B). Consistently, administration of baricitinib into IAV-infected mice from day 7 to day 11 after infection resulted in reduced KRT5^+^ alveolar area (Supplemental Figure 3, C–E). Interestingly, the formation of dysplastic KRT5^+^ cells was not affected in *Stat1^EKO^* mutant mice as compared with controls after IAV infection (Supplemental Figure 3, F–H), suggesting a dispensable role of *Stat1* in this process.

### Dysplastic KRT5^+^ cells exhibit elevated focal adhesion and YAP activity during their migration.

**Figure 3:**
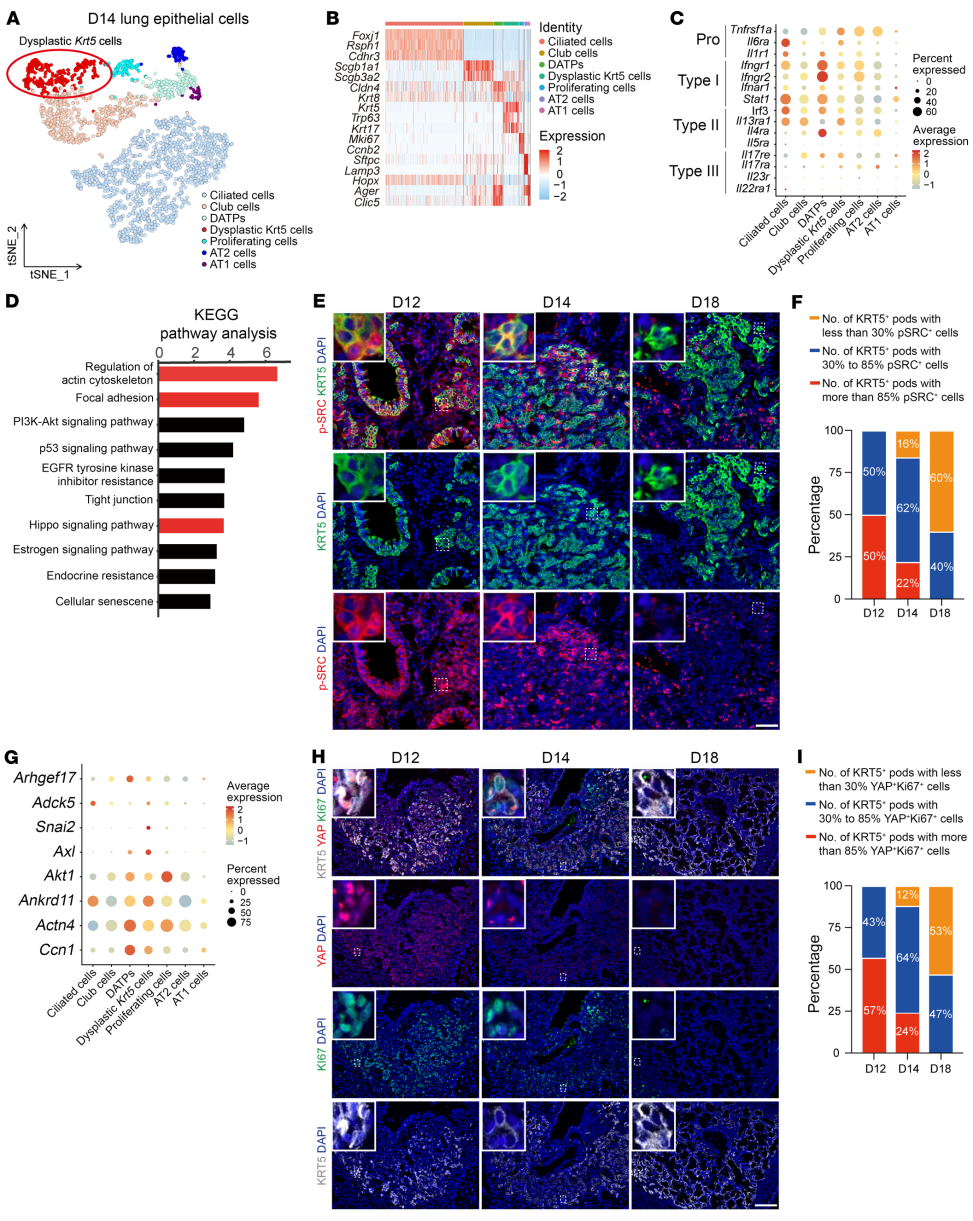
Dysplastic KRT5^+^ cells exhibited elevated FAK signaling and YAP activation during their expansion. (**A**) t-SNE clustering of lung epithelial cells from IAV-treated mice at day 14 after infection. (**B**) Heatmap showing marker gene expression in each cell cluster. (**C**) Dot plot showing expression of immune-related genes in each epithelial cell cluster. (**D**) KEGG analysis revealed highly activated pathways in KRT5^+^ cells compared with other airway epithelial cell populations. (**E** and **F**) Immunofluorescence images and quantification of the number of pods containing different percentages of p-SRC^+^KRT5^+^ cells at indicated time points after IAV infection. Data were collected from at least 4 mice at each time point and were counted as 1 biological replicate. Scale bar: 25 μm. (**G**) Dot plot showing expression of YAP target genes in lung epithelial cells from scRNA-Seq data. (**H** and **I**) Immunofluorescence images and quantification of number of pods containing different percentages of YAP^+^KI67^+^KRT5^+^ cells at indicated time points after IAV infection. Data were collected from at least 4 mice at each time point and were counted as 1 biological replicate. Scale bar: 50 μm.

To further investigate the intracellular signaling pathway by which IFN-γ regulates the formation of dysplastic KRT5^+^ cells, we performed single-cell RNA-sequencing (scRNA-Seq) assay on lung epithelial cells isolated from IAV-infected mice at day 14 after infection. The lung epithelial cells were classified into 7 cell clusters. These clusters consisted of secretory cells, ciliated cells, dysplastic KRT5^+^ cells, AT2 cells, AT1 cells, CLDN4^+^ intermediate cells, which are also named damage-associated transient progenitors (DATPs) or prealveolar type-1 transitional cell state (PATS) (26–28), and a cell population expressing high levels of cell-cycle–related genes, such as *Ki67* and *Ccnb2* (Figure 3, A and B, and Supplemental Figure 4A). We found that dysplastic KRT5^+^ cells, proliferating cells, and CLDN4^+^ intermediate cells were enriched in the expression of genes related to the type I cytokine signaling pathway, such as *Ifngr1*, *Ifngr2*, and *Stat1*, compared with genes related to type II and type III cytokines (Figure 3C). Kyoto Encyclopedia of Genes and Genomes (KEGG) pathway analysis revealed that dysplastic KRT5^+^ cells were enriched in the expression of genes associated with regulation of actin cytoskeleton, focal adhesion, and Hippo pathway (Figure 3D). In line with this, dysplastic KRT5^+^ cells expressed high levels of integrin and cell junction–related genes, such as *Itgb1*, *Itgb4*, *Itgb5*, *Itgb6*, *Cldn1*, and *Cldn4* (Supplemental Figure 4B). Because of these observations, we speculated that the actin cytoskeleton pathway and focal adhesion pathway, both of which can induce nuclear YAP activity via the FAK/c-Src (herein termed Src, the founding member of the Src family of nonreceptor protein tyrosine kinases) signaling axis (29), might play a role in regulating the expansion of dysplastic KRT5^+^ cells. Using an antibody specific to the active form of Src, phosphorylated at Tyr416 (30), we demonstrated that Src was widely activated in dysplastic KRT5^+^ cells at day 12 after infection, when these cells had just migrated into the alveoli (8, 18). As the KRT5^+^ cells expanded, the Src signal gradually decreased in dysplastic KRT5^+^ cells closer to the bronchoalveolar duct, while the dysplastic KRT5^+^ cells at the periphery of pods retained Src expression, consistent with the migration route of dysplastic KRT5^+^ cells that move from the distal airway to the alveoli (7, 18). By day 18 after infection, only a limited number of dysplastic KRT5^+^ cells were found expressing activated Src (Figure 3, E and F). By using a published dataset of YAP-regulated genes (31), we further demonstrated that canonical YAP target genes such as *Ccn1*, *Axl*, *Arfgeh17*, and *Snai2* were highly expressed in dysplastic KRT5^+^ cells (Figure 3G). Consistently, the nuclear YAP and Ki67 were expressed in a pattern resembling phosphorylated Src during dysplastic KRT5^+^ cell expansion, suggesting a causal link of YAP activation and dysplastic KRT5^+^ cell expansion (Figure 3, H and I). Notably, we also found that most of the SRC^+^ dysplastic KRT5^+^ cells expressed nuclear YAP (Supplemental Figure 4, C and D). Thus, these results demonstrate that the FAK/Src-YAP axis is highly activated during the expansion of dysplastic KRT5^+^ cells, prompting us to investigate their role in controlling this process.

### FAK/Src-YAP signaling axis is required for IFN-γ–mediated dysplastic remodeling.

**Figure 4:**
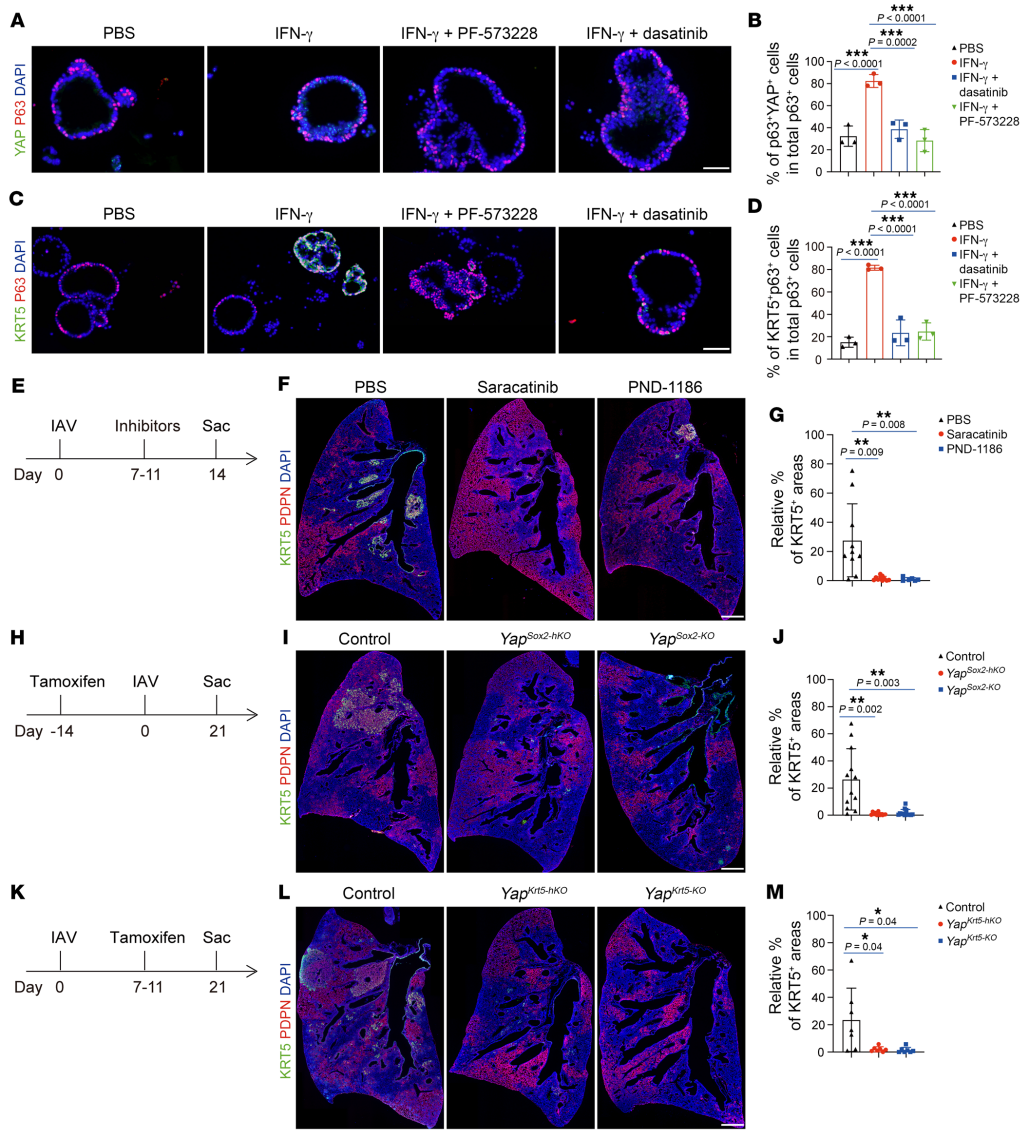
IFN-γ acts through the FAK/Src-YAP signaling axis to promote dysplastic KRT5^+^ cell expansion. (**A**) Immunofluorescence staining for YAP and p63 in cultured intrapulmonary p63^+^ cells treated with PBS, IFN-γ, IFN-γ and Src inhibitor (dasatinib), or IFN-γ and FAK inhibitor (PF-573228). Scale bar: 50 μm. (**B**) Quantification of percentages of p63^+^YAP^+^ cells in total p63^+^ cells (*n* = 3 technical replicates, experiment repeated twice). (**C**) Immunofluorescence staining for KRT5 and p63 in cultured intrapulmonary p63^+^ cells treated with PBS, IFN-γ, IFN-γ and Src inhibitor (dasatinib), or IFN-γ and FAK inhibitor (PF-573228). Scale bar: 50 μm. (**D**) Quantification of percentages of KRT5^+^p63^+^ cells in total P63^+^ cells (*n* = 3 technical replicates, experiment repeated twice). (**E**–**G**) Experiment design and quantification of percentages of dysplastic cell (KRT5^+^ PDPN^+^) areas in damaged alveolar areas (PDPN^–^ and KRT5^+^) in PBS-, Src inhibitor– (saracatinib), or FAK inhibitor–treated (PND-1186) mice at 14 dpi (*n* ≥ 6 mice per group). Scale bar: 500 μm. (**H**–**J**) Experiment design and quantification of percentages of KRT5^+^ dysplastic cell areas in damaged alveolar areas (PDPN^–^ and KRT5^+^) in control, *Yap*^Sox2–hKO^, and *Yap*^Sox2–KO^ mice at 21 dpi (*n* ≥ 12 mice per group). Scale bar: 500 μm. (**K**–**M**) Experiment design and quantification of percentages of KRT5^+^ dysplastic cell areas in damaged alveolar areas (PDPN^–^ and KRT5^+^) in control, *Yap*^Krt5–hKO^, and *Yap*^Krt5–KO^ mice at 21 dpi (*n* = 7 mice per group). Scale bar: 500 μm. *P < 0.05; **P < 0.01; ***P < 0.001. Error bars represent means ± SEM. One-way ANOVA (**B** and **D**); Brown-Forsythe and Welch’s ANOVA (**G**, **J**, and **M**).

Since IFN-γ is required for the formation of dysplastic KRT5^+^ cells and these cells exhibit high focal adhesion pathway activity during their migration, we hypothesized that IFN-γ may act through JAK to activate the FAK/Src-YAP pathway to mediate this dysplastic response. To evaluate the role of JAK in activating the FAK/Src-YAP pathway, we examined p-SRC and YAP expression in cultured primary intrapulmonary p63^+^ progenitor enriched cells and found increased p-SRC^+^ cells and YAP^+^ cells following IFN-γ treatment. Inhibition of JAK activity using baricitinib or fedratinib diminished this effect (Supplemental Figure 5, A–D). We also examined the total abundance of YAP using Western blot and found that IFN-γ treatment increased the total abundance of YAP, and this effect was diminished by adding baricitinib or fedratinib (Supplemental Figure 5E). Additionally, *Ifngr1* mutant mice exhibited a reduced number of nuclear YAP-expressing cells following IAV infection (Supplemental Figure 5, F–I). We next investigated the role of FAK/Src in this process. We found that in the presence of the FAK antagonist (PF-573228) or the Src antagonist (dasatinib), IFN-γ–mediated nuclear YAP translocation was also diminished (Figure 4, A and B). Similarly, IFN-γ treatment promoted cultured p63^+^ progenitor cells to transdifferentiate into KRT5^+^ cells, and this effect was inhibited by PF-573228 or dasatinib (Figure 4, C and D). Consistently, oral delivery of FAK antagonist (PND-1186) or Src antagonist (saracatinib) into IAV-infected mice from day 7 to day 11 after infection resulted in reduced KRT5^+^ lung areas compared with control mice receiving PBS (Figure 4, E–G).

We further tested the role of YAP in this process by crossing *Sox2^creERT2^* mice with *Yap^fl^* mice and generating *Yap* heterozygous (here after *Yap^Sox2–hKO^*) and homozygous mutant mice (here after *Yap^Sox2–KO^*). Tamoxifen was injected 14 days prior to IAV infection. The mutant mice showed normal ratios of airway and alveolar epithelial cells (Supplemental Figure 5, J–M). However, following IAV infection, the expansion of dysplastic KRT5^+^ cells was severely suppressed in both *Yap^Sox2–hKO^* and *Yap^Sox2–KO^* mutant lungs (Figure 4, H–J). To determine whether forced activation of YAP in SOX2^+^ progenitor cells promotes KRT5^+^ cell expansion in IAV-infected mice, we generated *Sox2^creERT2^;Mst1^fl/fl^;Mst2^–/–^* mutant mice (hereafter *Mst1/2*^Sox2–KO^), in which YAP is constitutively activated in SOX2 progenitor cells after tamoxifen induction (Supplemental Figure 6, A and B). After IAV infection, we observed a trend suggesting that constitutively activated YAP in SOX2^+^ progenitors promotes dysplastic KRT5^+^ cell formation, although it was not statistically significant (Supplemental Figure 6, C–E). We then assessed the requirement of YAP in expanding KRT5^+^ cells by generating *Krt5^creERT2^;Yap^fl/+^* (hereafter *Yap^Krt5–hKO^*) and *Krt5^creERT2^;Yap^fl/fl^* (hereafter *Yap^Krt5–KO^*) mice. Tamoxifen was injected from day 7 to day 11 after infection to induce CRE activity (Figure 4K). Similarly to what occurred with the phenotype of *Yap^Sox2–hKO^* mutant mice, the expansion of dysplastic KRT5^+^ cells was severely suppressed in both *Yap^Krt5–hKO^* and *Yap^Krt5–KO^* mutant lungs (Figure 4, L and M). Thus, in response to infection-induced IFN-γ signal, dysplastic KRT5^+^ cells activate FAK/Src-YAP signaling to proliferate and migrate to the injured lung alveoli.

### YAP is required for the long-term maintenance of dysplastic KRT5^+^ cells, and inhibition of YAP leads to their differentiation into club cells.

**Figure 5:**
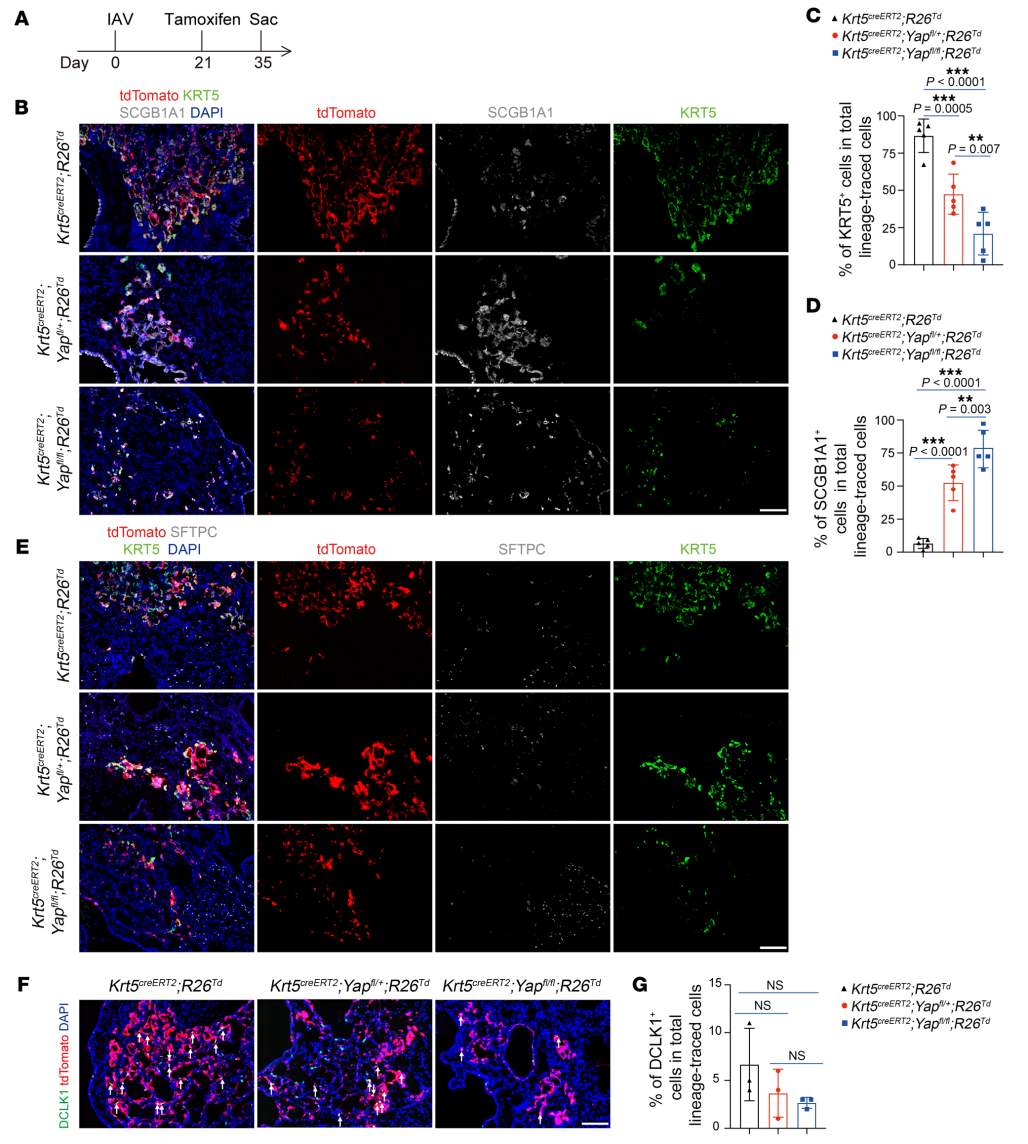
YAP is required for the maintenance of dysplastic KRT5^+^ cells. (**A**) Illustration of strategy to trace dysplastic KRT5*^+^* cell fate while inactivating *Yap*. (**B**–**D**) Immunofluorescence images and quantification of percentages of lineage-traced KRT5^+^ dysplastic cells or SCGB1A1^+^ club cells in *Krt5^creERT2/+^;R26^Td^*, *Krt5^creERT2/+^;Yap^fl/+^;R26^Td^*, and *Krt5^creERT2/+^;Yap^fl/fl^;R26^Td^* mice at 35 dpi (14 days after tamoxifen injection) (*n* = 5 mice per group). Scale bar: 50 μm. (**E**) No *Krt5*^creERT2^ lineage-traced cells were found expressing SFTPC in *Krt5^creERT2/+^;R26^Td^*, *Krt5^creERT2/+^;Yap^fl/+^;R26^Td^*, and *Krt5^creERT2/+^;Yap^fl/fl^;R26^Td^* mice at 35 dpi. Data are representative of sections from 3 mice. Scale bar: 50 μm. (**F**) Immunofluorescence staining of DCLK1 in *Krt5*^creERT2/+^;R26^Td^, *Krt5*^creERT2/+^;*Yap*^fl/+^;R26^Td^, and *Krt5*^creERT2/+^;*Yap*^fl/fl^;R26^Td^ mice at 35 dpi (arrows indicate lineage-labeled DCLK^+^ tuft cells). Scale bar: 50 μm. (**G**) Quantification of percentages of tuft cells in total lineage-traced cells (*n* = 3 mice per group). **P < 0.01; ***P < 0.001. Error bars represent means ± SEM. One-way ANOVA (**C**, **D**, and **G**).

The persistence of alveolar KRT5^+^ cells impedes functional lung regeneration and chronically affects lung function (11). To determine whether YAP is also required for the long-term maintenance of dysplastic KRT5^+^ cells in the alveoli, we introduced R26-tdtomato reporter into a *Yap^Krt5–hKO^* and *Yap^Krt5–KO^* mutant mouse background. This allowed us to trace dysplastic KRT5^+^ cells while assessing the possible cell-fate conversion that may be induced by *Yap* inactivation. Tamoxifen was administered at day 21 after infection, when most of KRT5^+^ cells had migrated into the alveoli and no longer expressed Ki67, and the mice were analyzed at 14 days after tamoxifen administration (Figure 5A). Immunofluorescent analysis showed that most of the lineage-traced cells in control animals continued to express KRT5, with only a few cells losing KRT5 expression and instead expressing SCGB1A1 (Figure 5B). In contrast, in *Yap* mutant mice, we observed an increase in the proportion of lineage-traced cells expressing club cell markers, including SCGB1A1, ITGb4, and SCGB3A2 (Figure 5B and Supplemental Figure 6, F and G), while the percentages of cells expressing KRT5 decreased. Interestingly, this phenotype was also shown to be YAP dose dependent (Figure 5, C and D). We did not observe any lineage-traced cells expressing surfactant-associated protein C (SFTPC) in either control or *Yap* mutant mice (Figure 5E). The percentage of DCLK1^+^ tuft cells in total lineage-traced cells was not affected upon *Yap* inactivation (Figure 5, F and G). Intriguingly, though *Yap* deletion affects the maintenance of dysplastic KRT5^+^ cells, the lung fibrosis was not ameliorated (Supplemental Figure 6H). Thus, we provide a strategy for converting persistent KRT5^+^ cells into distal club-like cells.

### IFN-γ promotes human AT2 cells to transdifferentiate into dysplastic cells.

**Figure 6:**
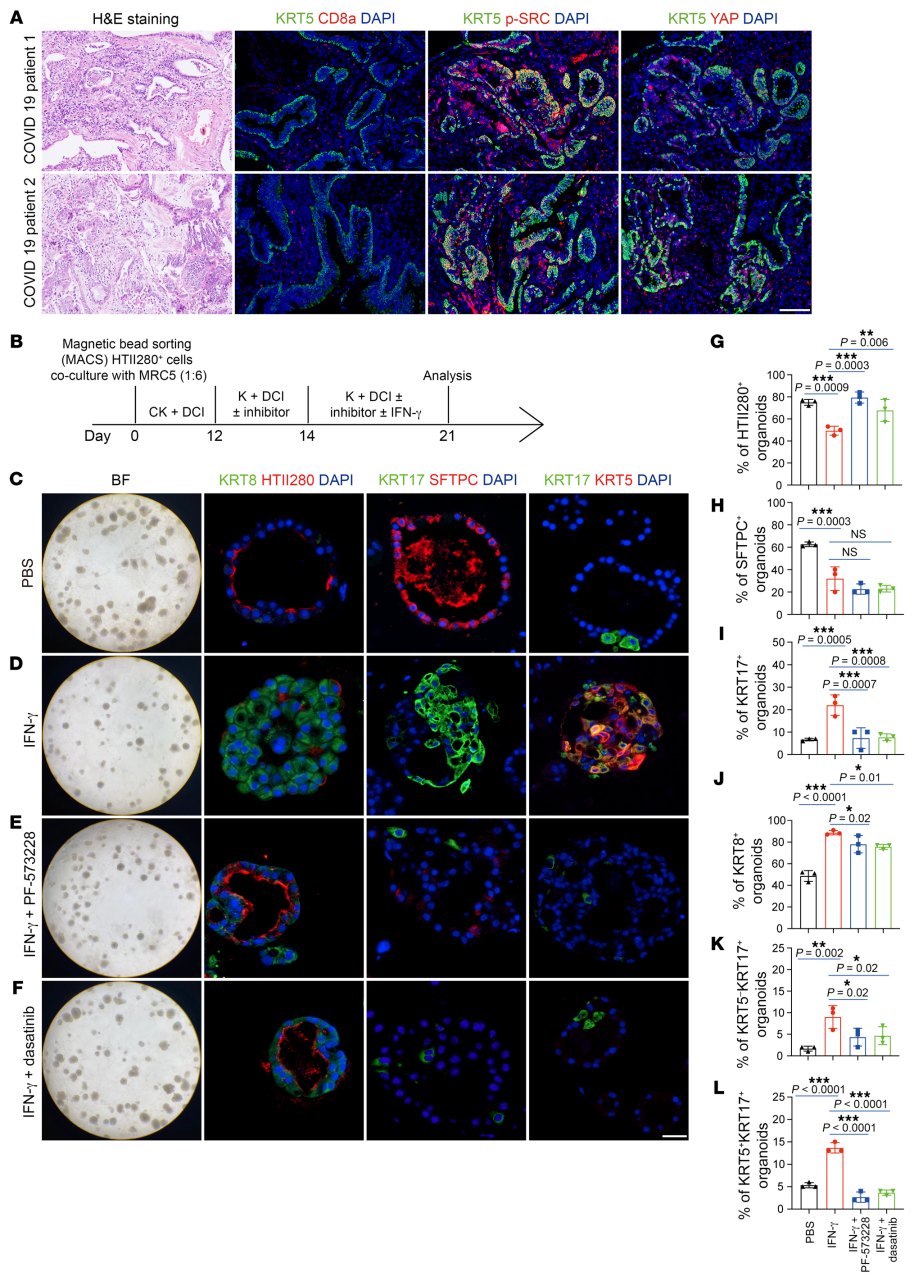
IFN-γ treatment converted human AT2 cells into dysplastic KRT5^+^ cells. (**A**) H&E staining and immunofluorescence staining for CD8a, p-SRC, YAP, and KRT5 on lung sections from COVID-19 patients. Scale bar: 50 μm. (**B**) Illustration of human AT2 organoid culture experiment. (**C**–**F**) Bright-field and immunofluorescence images of human AT2 organoids treated with PBS, IFN-γ, IFN-γ and Src inhibitor (dasatinib), or IFN-γ and FAK inhibitor (PF-573228). Scale bar: 25 μm. (**G**–**L**) Quantification of percentages of HTII-280–, SFTPC-, KRT8-, KRT17-, and KRT5-expressing organoids in total human AT2 organoids treated with PBS, IFN-γ, IFN-γ and Src inhibitor (dasatinib), or IFN-γ and FAK inhibitor (PF-573228) (*n* = 3 technical replicates, experiment repeated twice). *P < 0.05; **P < 0.01; ***P < 0.001. Error bars represent means ± SEM. One-way ANOVA (**G**–**L**).

Unlike in mice, in which the dysplastic KRT5^+^ cells are derived from intrapulmonary p63^+^ cells, in humans, AT2 cells can transdifferentiate into dysplastic KRT5^+^ cells during lung injury or disease (32). To investigate whether IFN-γ signaling also mediates dysplastic cell formation in human lungs, we first analyzed published scRNA-Seq data of COVID-19 lungs (33). We included AT2 cells, AT1 cells, KRT8^+^ intermediate cells, and basal cells in our analysis. Monocle 3 analysis suggested that human AT2 cells differentiate into AT1 cells and KRT8^+^ intermediate cells through different trajectories and that KRT8^+^ intermediate cells may further differentiate into basal cells (Supplemental Figure 7, A and B). This observation is consistent with a recent report that in human lungs, KRT8^+^ intermediate cells can serve as precursors for dysplastic KRT5^+^ cells (32). Gene Set Enrichment Analysis (GSEA) revealed that these KRT8^+^ intermediate cells exhibited high activity in response to IFN-γ, focal adhesion, and the actin cytoskeleton pathway compared with AT2 cells (Supplemental Figure 7, C–E). Using lung sections from COVID-19 patients, we observed CD8^+^ T cells appearing in the KRT5^+^ lung area and p-SRC and nuclear YAP expression in KRT5^+^ cells (Figure 6A). To further investigate whether IFN-γ can promote the transdifferentiation of human AT2 cells into dysplastic KRT5^+^ cells through the FAK/Src-YAP pathway, we isolated primary AT2 cells from human lung tissues using magnetic bead–based selection with HTII-280 antibody (Supplemental Figure 7, F and G). The sorted cells were cocultured with MRC5 cells, a human fetal lung fibroblast cell line, as feeders to promote the organoid formation. Recombinant human IFN-γ was added to the culture medium on day 14 when single AT2 cells had grown into sphere-like organoids, and organoids were collected at day 21 for analysis (Figure 6B). Immunofluorescent analysis showed that following IFN-γ treatment, there was a decrease of organoids expressing the AT2 cell marker, including SFTPC and HTII-280, whereas the organoids expressing KRT8, KRT17, or KRT5 increased (Figure 6, C and D). Interestingly, some KRT8^+^ cells also expressed HTII-280 (Supplemental Figure 7, H–K). Supplementation of IFN-γ–treated human AT2 organoids with Src antagonist (dasatinib) or FAK antagonist (PF-573228) prevented their transdifferentiation into KRT8^+^, KRT17^+^, or KRT5^+^ dysplastic cells and partially reversed the expression of HTII-280, but the expression of SFTPC was not reversed by either of these 2 inhibitors (Figure 6, E and F). We also confirmed the expression of p-SRC and YAP in IFN-γ–treated human lung organoids (Supplemental Figure 7, L–O). These observations were supported by detailed quantification using cell-type–specific markers (Figure 6, G–L). Collectively, these findings in humans are consistent with our findings in mice and are suggestive of a potential role of IFN-γ and FAK/Src-YAP signaling in controlling viral pneumonia–induced dysplastic remodeling in human lungs.

## Discussion

Several mechanisms have been proposed for understanding the activation of dysplastic remodeling in viral-infected lungs (8, 10, 23). However, why viral infection, as opposed to other types of injury, is particularly effective in triggering this dysplastic response remains unexplained. Our findings reveal that a viral infection–induced inflammatory niche is critical for the robust expansion of KRT5^+^ dysplastic cells. Inactivation of IFN-γ signaling reduced the formation of dysplastic cells in IAV- but not bleomycin-injured lungs, suggesting that the dysplastic cell formation is differentially regulated between these 2 injury models. This is consistent with recent studies that show intrapulmonary p63^+^ cells are barely activated upon bleomycin challenge (34, 35). Previous studies showed that blocking IFN-γ signaling during respiratory viral infection limits tissue injury and reduces lung pathology (36–39). The results showing that epithelial *Ifngr1* mutant mice show improved lung recovery after IAV infection, indicated by increased AT2 cell formation and improved lung capacity, suggest that dysplastic cell formation contributes to IFN-γ–induced lung pathology. It is worth noting that, in addition to T cells, we also observed more monocyte-derived alveolar macrophages in IAV- than bleomycin-injured lungs; these macrophages are extensively involved in tissue injury repair (20, 40). A recent study suggests that IL-22 can also act on lung epithelial cells and promote dysplastic cell formation (41), indicating that infection-induced inflammatory factors may work in a cooperative manner to control lung dysplastic remodeling. These complementary results highlight the role of an injury-induced inflammatory signal in promoting aberrant tissue regeneration and provide a perspective for understanding the frequent occurrence of dysplastic remodeling following severe viral pneumonia.

Cell migration requires dynamic rearrangement of the actin cytoskeleton. Our findings demonstrate that dysplastic KRT5^+^ cells exhibit high activity of the actin cytoskeleton and focal adhesion pathways during their migration. Similarly to the transition from AT2s to AT1s (42), intrapulmonary p63^+^ cells change their small, wedged cell shape into an extended and flattened cell shape when transformed into dysplastic KRT5^+^ cells, indicating extensive cytoskeletal remodeling. As a critical downstream effector of the actin cytoskeleton and focal adhesion signaling pathways, the nuclear YAP signal is highly activated during this process. Interestingly, the KRT8^+^ intermediate cells in IAV-infected lungs also express high levels of genes related to IFN-γ and YAP signaling. Using multiple transgenic mouse lines, we demonstrate that high YAP activity is required for dysplastic KRT5^+^ cell initiation, migration, and maintenance. Heterozygous deletion of YAP in intrapulmonary p63^+^ progenitor cells or migrating dysplastic cells prevents their expansion, while in persistent dysplastic cells, it converts them into distal club–like cells. As distal club cells can function as progenitor cells to generate AT2s in various conditions (1, 5, 28), this finding suggests a promising strategy for converting dysplastic KRT5^+^ cells into distal lung progenitor cells. It is worth noting that in addition to regulating KRT5^+^ cells, hippo signaling has also been shown to regulate AT2 cells to AT1 cell differentiation (43–45). During lung injury, AT2 cells are important lung progenitor cells for repairing of an alveolar area with mild to moderate injury (46). Future studies to uncover the specific mechanism that regulates hippo signaling activation across different cell types will provide new therapeutic strategies for targeting hippo signaling for promoting lung regeneration.

The ectopic expansion of dysplastic KRT5^+^ cells in lung alveoli has been reported in patients after influenza or COVID-19 infection. Unlike mouse AT2 cells, which can only differentiate into AT1 cells, human AT2 cells can also differentiate into dysplastic cells upon injury. The finding that dysplastic cells in COVID-19 patient lungs express p-SRC and nuclear YAP signal and that IFN-γ promotes human AT2 cell transdifferentiate into dysplastic cells in a FAK/Src-dependent manner suggests that IFN-γ–mediated activation of the FAK/Src-YAP signaling axis may also regulate dysplastic alveolar remodeling in viral-infected human lungs. Overall, our findings highlight the role of injury-induced inflammatory signals in the activation of alveolar remodeling and uncover a critical role of YAP in controlling dysplastic cell expansion and maintenance.

## Methods

### Sex as a biological variable.

Our study examined male and female animals, and similar findings are reported for both sexes. Human samples were obtained from both male and female subjects.

### Mice.

*Shh^cre^*, *Ifngr1^fl/fl^*, *Sox2^creERT^*, *Krt5^creERT^*, *R26-tdTomato(R26-tdT)*, *Yap^fl/fl^*, and *Mst1^fl/fl^;Mst2^–/–^* mice have all been described previously (10, 17, 20, 34, 47–49). *Stat1^fl/fl^* mice (T051946) were purchased from GemPharmatech. All mice were bred on the C57BL/6 background and kept in specific pathogen–free conditions with a 12-hour light/12-hour dark cycle.

### Influenza- and bleomycin-injury models.

Influenza A/H1N1/Puerto Rico/8/34 (PR8) was propagated in embryonated chicken eggs. Male (8–10 weeks) and female (10–12 weeks) mice were anesthetized by intraperitoneal injection of 200 mg/kg sodium pentobarbital and then intratracheally administered with 100 PFU PR8 in 40 μL sterile PBS (pH = 7.4). For bleomycin treatment, male (6–8 weeks) and female (8–10 weeks) mice were anesthetized by isoflurane and intranasally administered with 1 mg/kg bleomycin (Hushi, XW90419342) in 40 μL sterile PBS once a week for 4 weeks.

### Tamoxifen treatment.

Tamoxifen was completely dissolved in anhydrous ethanol at a concentration of 100 mg/mL (need vortex and instantaneous heating), then diluted in corn oil to 10 mg/mL. Mice were treated with 100 mg/kg tamoxifen (ABCONE, T56488) by intraperitoneal injection at indicated time points.

### Inhibitor treatment.

Src inhibitor saracatinib (20 mg/kg/time, Selleck, S1006-100 mg), FAK inhibitor PND-1186 (150 mg/kg/time, Selleck, S7653-200 mg), or JAK1/JAK2 inhibitor baricitinib phosphate (10 mg/kg/time, MCE, HY-15315A-50 mg) was delivered via oral gavage from day 7 to day 11 after infection. Mice were sacrificed at day 14 after infection.

### MVV detection.

Mice were anesthetized by intraperitoneal injection of 200 mg/kg sodium pentobarbital and then MVV was detected using the RoVent Advanced Small Animal Ventilator (Kent Scientific).

### Pulmonary function test.

Lung-function parameters were measured using the EMMS eSpira Forced Manoeuvers System. Mice were anesthetized before an endotracheal cannula was inserted into their trachea. The dynamic compliance results were obtained from the resistance and compliance test. FVC results were obtained from the pressure volume test.

### Histology staining.

Mice were sacrificed with CO_2_, and lungs were inflated with 4% paraformaldehyde (PFA) and fixed overnight, followed by 3 washes with PBS. Human lung tissues were fixed in 4% PFA overnight, followed by 3 washes with PBS. Organoids were collected and fixed with 4%PFA for 30 minutes at 4°C. Lungs tissues and organoids were then prepared for paraffin (8 μm) or cryo (10 μm) sectioning. Goblet cells were stained using a Periodic Acid–Schiff (PAS) Staining Kit (MilliporeSigma). Collagen fibers were stained using the Picro Sirius Red Stain Kit (Phygene). To quantify PAS-positive area and fibrosis area, the left lung lobes were imaged using Olympus BX51 and an image mosaic was created using ImageJ software (NIH). The PAS-positive areas and fibrosis areas were measured using ImageJ.

### Immunostaining.

The following primary antibodies were used at the indicated concentrations for immunofluorescence staining: goat anti-SCGB3A2 polyclonal antibody (5 μg/mL) (R&D Systems, AF3465), rat anti-ITGB4 monoclonal antibody (5 μg/mL) (BioLegend, 123615), rat anti-F4/80 monoclonal antibody (5 μg/mL) (BioLegend, 111603), rabbit anti-CCR2 monoclonal antibody (5 μg/mL) (Abcam, ab216863), rabbit anti-KRT5 polyclonal antibody (5 μg/mL) (BioLegend, 905504), chicken anti-KRT5 polyclonal antibody (5 μg/mL) (BioLegend, 905903), mouse anti-SCGB1A1 monoclonal antibody (5 μg/mL) (Santa Cruz Biotechnology Inc., sc-365992), rabbit anti-CCSP polyclonal antibody (5 μg/mL) (Seven Hills, WRAB-3950), mouse anti-FOXJ1 monoclonal antibody (5 μg/mL) (Invitrogen, 14-9965-82), hamster anti-PDPN monoclonal antibody (5 μg/mL) (Invitrogen, 14-5381-82), rat anti-CD4 monoclonal antibody (5 μg/mL) (BioLegend, 100427), rat anti-CD8a monoclonal antibody (5 μg/mL) (BioLegend, 100707), mouse anti-NK1.1 monoclonal antibody (5 μg/mL) (BioLegend, 108729), rabbit anti-DCAMKL1 polyclonal antibody (5 μg/mL) (Abcam, ab31704), rabbit anti-proSPC polyclonal antibody (5 μg/mL) (MilliporeSigma, AB3786), rabbit anti-phospho-Src(Tyr416) polyclonal antibody (5 μg/mL) (Cell Signaling Technology, 2101S), rabbit anti-YAP polyclonal antibody (5 μg/mL) (Cell Signaling Technology, 4912S), mouse anti-KI67 monoclonal antibody(5 μg/mL) (BioLegend, 151204), rabbit anti-KI67 monoclonal antibody (5 μg/mL) (Cell Signaling Technology, 9129S), mouse anti-P63 monoclonal antibody (5 μg/mL) (Abcam, ab735), mouse anti-KRT17 monoclonal antibody (5 μg/mL) (Santa Cruz Biotechnology Inc., sc-393002), rat anti-KRT8 polyclonal antibody (5 μg/mL) (DSHB, Troma-1), mouse anti-human HTII-280 (5 μg/mL) (Terrace Biotech, TB-27AHT2-280), and mouse anti-human CD8a monoclonal antibody (5 μg/mL) (Invitrogen, 12-0088-42). The following secondary antibodies were from Jackson Immunoresearch: Cy3-conjugated goat anti-rabbit IgG (5 μg/mL) (code 111-165-003), FITC-conjugated goat anti-rabbit IgG (5 μg/mL) (code 111-095-003), Cy3-conjugated goat anti-mouse IgG (5 μg/mL) (code 115-165-003), FITC-conjugated goat anti-mouse IgG (5 μg/mL) (code 115-095-003), Cy3-conjugated goat anti-rat IgG (5 μg/mL) (code 112-125-003), and FITC-conjugated goat anti-rat IgG (5 μg/mL) (code 112-545-003). The following secondary antibodies were from Abcam: Alexa Fluor 555–conjugated donkey anti-goat IgG H&L (5 μg/mL) (ab150130), Alexa Fluor 647–conjugated goat anti-chicken IgY (5 μg/mL) (ab150171), Alexa Fluor 488–conjugated goat anti-chicken IgY (5 μg/mL) (ab150169), and Alexa Fluor 405–conjugated goat anti-chicken IgY (5 μg/mL) (ab175674). The following secondary antibodies were from Invitrogen: Alexa Fluor 488–conjugated goat anti-Syrian hamster IgG (5 μg/mL) (A-21110) and Alexa Fluor 546–conjugated goat anti-Syrian hamster IgG (5 μg/mL) (A-21111). DAPI solution (1 mg/mL) (Invitrogen) was used to counterstain nuclei. Images were acquired by Olympus BX51. Data were analyzed with ImageJ (NIH).

YAP immunofluorescent staining was performed using TSA fluorescein kits. The following antibodies were used: rabbit anti-YAP polyclonal antibody (5 μg/mL) (Cell Signaling Technology, 4912S) and goat anti rabbit IgG (H+L) HRP (5 μg/mL) (Invitrogen, 31430). Alexa Fluor 488 tyramide reagent (Invitrogen, B40953) was used to amplify the signal.

### Quantification of KRT5^+^ lung area.

To quantify KRT5^+^ or PDPN^–^ area, the left lung lobes were sectioned and stained for KRT5 and PDPN antibodies, then were imaged by Olympus BX51, and an image mosaic was created using ImageJ software. The KRT5^+^ or PDPN^–^ areas were measured using outline spline in the measure menu of Axiovision 4.8.

### Quantification of pods with different expression levels of YAP, SRC, and KI67.

To quantify number of different types of pods in lung, the left lung lobes were sectioned and stained for KRT5 and p-SRC antibodies or KRT5 and KI67 and YAP antibodies, then were imaged using Olympus BX51, and an image mosaic was created using ImageJ software. We defined more than 85% of cells as KRT5^+^p-SRC^+^ cells or KRT5^+^KI67^+^YAP^+^ cells as high pods, 30% to 85% of cells as KRT5^+^p-SRC^+^ cells or KRT5^+^KI67^+^YAP^+^ cells as medium pods, and less than 30% of cells as KRT5^+^p-SRC^+^ cells or KRT5^+^KI67^+^YAP^+^ cells as low pods.

### Western blot.

Organoids were collected and washed in PBS to remove Matrigel and then lysed in RIPA (50 mM tris-HCl [pH 8.0], 150 mM NaCl, 0.1% SDS, 0.15% Na-Deoxycholate, 1% NP-40, and 2 mM EDTA [pH 8.0]) containing protease inhibitors (MCE, 317717) and phosphatase inhibitor PhosSTOP (Roche, 04906845001). Protein content was quantified by standard bicinchoninic acid (BCA) assay using the Omni-Easy BCA Protein Assay Kit (epizyme, ZJ102). Equal protein amounts (10 μg/sample) were run on 4.5%–10% bis-tris protein gels (epizyme, PG112) and transferred to PVDF Transfer Membranes (Thermo Scientific, 88518). Membranes were blocked in 5% milk solution for 1 hour at room temperature and probed overnight at 4°C with YAP antibodies (Cell Signaling Technology, 4912S) in 1:1,000 dilution and β-actin antibody (Cell Signaling Technology, 4967) in 1:5,000. After 3 washes in 1× Tris-buffered saline with 0.1% Tween 20 detergent (TBST) buffer (10 minutes each), membranes were incubated with peroxidase-labeled anti-rabbit (Invitrogen, 31460) secondary antibody (1:5,000) and peroxidase-labeled anti-mouse (Invitrogen, 31430) secondary antibody (1:5,000) for 1 hour at room temperature. After subsequent washes in 1× TBST buffer, the Enhanced Chemiluminescence Substrate Kit (Thermo Fisher Scientific, 32132) was used for band detection according to the manufacturer’s instructions. Membranes were imaged on the ChemiDoc MP Imaging System (Bio-Rad).

### Sircol collagen assay.

Collagen quantification was performed using the Sirius Red Total Collagen Detection Kit following the manufacturer’s protocol (Chondrex, 9062). Briefly, the left lung was homogenized and digested overnight at 4°C in 2 mL 0.05 M acetic acid with 0.1% v/m pepsin and centrifuged at 18,400*g* for 30 minutes. The supernatant was collected, and 0.5 mL Sircol dye was incubated with 100 μL of the supernatant for 20 minutes. This sample was centrifuged at 9,600*g* for 3 minutes, and the resulting pellet was washed in the acid salt wash buffer and resuspended in 250 μL extraction buffer. Absorbance was measured at a wavelength of 530 nm in a microplate reader. The collagen content was determined by comparing it to a standard curve, represented as mg/mL of left lung homogenate.

### BALF and lung homogenate cytokine panel.

Mice were euthanized by intraperitoneal injection of a lethal dose of sodium pentobarbital, after which a catheter was introduced into the trachea. A 1 mL syringe was loaded with 1 mL of sterile balanced salt solution with protease inhibitors, and then BALF was collected. This was repeated 3 times. Lavage fluid was centrifuged for 7 minutes at 400*g* and 4°C. Supernatant was collected and stored at –80°C. Mice were euthanized and whole lung was collected in PBS with protease inhibitors, then homogenate, and centrifuged at 18,400*g* for 30 minutes. The supernatant was collected and stored at –80°C. We detected cytokines using the Mouse Inflammation Panel (13-plex) with V-bottom Plate Flow Cytometry Kit following the manufacturer’s protocol (BioLegend, 740446). Briefly, samples were incubated with beads and shaken at 800 rpm on a plate shaker for 2 hours at room temperature. Samples were washed before being incubated with detection antibodies and shaken at 800 rpm on a plate shaker for 1 hour at room temperature. SA-PE was added directly, and samples were shaken at 800 rpm on a plate shaker for 0.5 hours at room temperature. Beads were washed and resuspended by pipetting and samples were read on flow cytometer BD Fortessa (BD Biosciences). The assay FCS files were analyzed using BioLegend’s LEGENDplex data analysis software, version 8.0.

### Flow cytometry.

Mice were sacrificed with CO_2_ and perfused with 5 mL of cold PBS to eliminate blood. Whole lungs were digested by gentle shaking in lysis buffer containing DMEM, 225 U/mL collagenase type II, 25 U/mL DNase I, 5% FBS, 2 mM MgCl_2_, 2 mM CaCl_2_, and 20 mM HEPES for 60 minutes at 37°C, and then mechanically dissociated using GentleMACS C tubes (Miltenyi Biotec), followed by straining through a 70 μm filter resuspended in 40% Percoll, underlining with 66% Percoll. We centrifuged at 700*g* for 20 minutes and set deceleration (DEC) to 0” in this step. Hematopoietic cells were isolated from the interphase for analysis. The single-cell suspensions from above were pelleted, suspended (PBS), aliquoted at approximately 1 × 10^6^ cells per tube, and stained with Fc blocking antibody (5 mg/mL, BD) and the Live/Dead Fixable Dead Cell Stain Kit (Invitrogen) at room temperature for 10 minutes. The cells were washed and then incubated with antibody cocktail for 45 minutes at 4°C. The following antibodies were used: Pacific blue–conjugated rat anti-CD4 monoclonal antibody (5 μg/mL) (BioLegend, 100427), PE-conjugated rat anti-CD8a monoclonal antibody (5 μg/mL) (BioLegend, 100707), Alexa Fluor 700–conjugated mouse anti-NK1.1 monoclonal antibody (5 μg/mL) (BioLegend, 108729), Apc-cy7–conjugated rat anti-CD90.2 monoclonal antibody (5 μg/mL) (BioLegend, 105327), FITC-conjugated rat anti-CD45 monoclonal antibody (5 μg/mL) (BioLegend, 103122), Alexa Fluor 700–conjugated mouse anti-CD11b monoclonal antibody (5 μg/mL) (BioLegend, 111222), PE-conjugated rat anti-F4/80 monoclonal antibody (5 μg/mL) (BioLegend, 111603), and APC-conjugated rat anti-Ly6c monoclonal antibody (5 μg/mL) (BioLegend, 128015). Absolute counting beads were used to count cell numbers (Invitrogen, C36950). Lymphocytes were sorted as AmCyan^–^FITC^+^Apc-cy7^+^ cells. NK cells were sorted as AmCyan^–^FITC^+^Alexa Fluor 700^+^ cells, CD4^+^ T cells were sorted as AmCyan^–^FITC^+^Apc-cy7^+^Pacific blue^+^ cells, and CD8^+^ T cells were sorted as AmCyan^–^FITC^+^Apc-cy7^+^PE^+^ cells. Alveolar macrophages were sorted as AmCyan^–^FITC^+^Apc-cy7^+^Alexa Fluor 700^+^PE^+^ cells, and monocyte-derived inflammatory macrophages were sorted as AmCyan^–^FITC^+^Apc-cy7^+^Alexa Fluor 700^+^APC^+^ cells. Samples were analyzed using BD Fortessa (BD Biosciences). Data were analyzed with FlowJo software, version 10.4

### Mouse tissue digestion and cell sorting.

Mice were sacrificed using CO_2_ at 14 days post infection (dpi) after IAV challenge and transcardially perfused with 5 mL of cold PBS. Whole lungs were digested by gentle shaking in lysis buffer containing DMEM, 2.5 U/mL dispase II, 225 U/mL collagenase type I, 25 U/mL DNase I, 5% FBS, 2 mM MgCl_2_, 2 mM CaCl_2_, and 20 mM HEPES for 30 minutes at 37°C. Cell suspension was filtered through a 70 μm strainer, and red blood cells were removed using RBC lysis buffer (BioLegend, 420301). The single-cell suspensions from above were pelleted and stained for indicated antibodies.

For scRNA-Seq, the following antibodies were used: FITC-conjugated rat anti-CD45 (5 μg/mL) (BioLegend, 103107), FITC- conjugated rat anti-CD31 (5 μg/mL) (BioLegend, 102405), and APC-conjugated rat anti-EPCAM (5 μg/mL) (BioLegend, 118214). DAPI solution (1 mg/mL) (Invitrogen) was used to counterstain nuclei. Anti-FITC microbeads (Miltenyi Biotec) were used to clear endothelial cells and immune cells by AutoMACS Pro. Target cells were sorted as DAPI^–^FITC^–^APC^+^ using BD FACSAria III.

For sorting LNEP cells and fibroblast, the following antibodies were used: FITC-conjugated rat anti-CD45 (5 μg/mL) (BioLegend, 103107), FITC-conjugated rat anti-CD31 (5 μg/mL) (BioLegend, 102405), APC-cy7–conjugated rat anti-EPCAM (5 μg/mL) (BioLegend, 118217), APC-conjugated rat anti-CD200 (5 μg/mL) (BioLegend, 123809), and PE-cy7–conjugated rat anti-integrin β4 (5 μg/mL) (BioLegend, 123615). DAPI solution (1 mg/mL) (Invitrogen) was used to counterstain nuclei. Anti-FITC microbeads (Miltenyi Biotec) were used to clear endothelial cells and immune cells by AutoMACS Pro. LNEP cells were sorted as DAPI^–^FITC^–^APC-cy7^+^APC^+^PE-cy7^+^ cells, and fibroblasts were sorted as DAPI^–^FITC^–^APC-cy7^–^ cells.

### Mouse organoid culture.

LNEP cells and fibroblasts (20,000:50,000) were cocultured in Pneumacult-ALI Medium (STEMCELL Technologies, 5001) diluted 1:1 in growth factor–reduced Matrigel (Corning-Biocoat, 356231) and seeded in trans wells (TCS002024). Pneumacult-ALI Medium with 5% FBS was added in the first 7 days to form spheres; 10 μM Y27632 (MCE, HY-10583) was added at the first 2 days, and culture medium was replenished every other day. For IFN-γ treatment, 100 ng/mL mouse recombinant IFN-γ (novoprotein, C746) or PBS was added at day 8, and organoids were collected at day 11 for analysis. For inhibitor treatment, cells were pretreated with indicated inhibitors at day 7. Twenty-four hours later, fresh medium containing IFN-γ and indicated inhibitors were replenished and organoids were maintained for another 72 hours. The following molecules were used for LNEP organoid treatment: 10 μM JAK1/JAK2 inhibitor fedratinib (MCE, HY-10409), 10 μM JAK1/JAK2 inhibitor baricitinib (MCE, HY-15315), 10 μM FAK inhibitor PF-573228 (MCE, HY-10461), and 10 μM Src inhibitor dasatinib (MCE, HY-10181).

### scRNA-Seq and analysis.

Sorted epithelial cells were used to perform scRNA-Seq with the 10x Genomics Chromium Platform at 14 dpi. FASTQ files were processed using the Cell Ranger pipeline. For further downstream analysis, R package Seurat (version 4.2.0) was used. Low-quality cells (with fewer than 200 or more than 7,500 genes and with more than 10% mitochondrial transcripts) were removed. ScaleData(), RunPCA(), FindNeighbors(), FindClusters() and RunTSNE() functions were used to cluster and visualize cells. Cell annotation was achieved according to the marker gene expression. Immune receptor expression was plotted using the Dotplot() function. The upregulated expression gene set was used to perform KEGG enrichment analysis using the clusterProfiler R package after removal of ribosomal genes. Visualization of YAP downstream gene expression was shown using Nebulosa (version 1.8.0) R package. The trajectory analysis was inferred using the R package Monocle 3 (version 1.0.0) by importing the counts from the Seurat object. For reanalysis of human RNA-Seq data, only data from COVID-19–infected lungs were used.

### Human AEC2 sorting.

Human lung tissue was dissected into 1 cm^3^ pieces and washed with 50 mL cold PBS, then dissected into smaller pieces (1–2 mm^3^). Human lung pieces were digested by gentle shaking in lysis buffer containing DMEM, 2.5 U/mL dispase II, 225 U/mL collagenase type I, 100 U/mL DNase I, 5% FBS, 2 mM MgCl_2_, 2 mM CaCl_2_, and 20 mM HEPES for 60 minutes at 37°C. The tissue homogenate was filtered through a 70 μm strainer, and red blood cells were removed using RBC Lysis Buffer (BioLegend, 420301). The single-cell suspensions from above were pelleted and resuspended in PBS buffer containing 5% FBS and anti-human HTII-280 (5 μg/mL) (Terrace Biotech, TB-27AHT2-280). Cells were stained for 30 minutes at 4°C and further sorted using anti-mouse IgM microbeads (Miltenyi Biotec) with AutoMACS Pro.

### Human organoid culture.

Human AEC2 and MRC5 cells (ATCC, CCL-171) (5,000:30,000) were diluted in equal volumes of Matrigel, seeded in Transwells (TCS002024), and solidified at 30 minutes at 37°C. CK+DCI medium is composed of the following components: IMDM (Thermo Fisher Scientific, 12440053), Ham’s F12 medium (CellGro, 10-080-CV), 3 μM CHIR99021 (Sigma-Aldrich, SML1046-5MG), 10 ng/mL rhKGF (Novoprotein, CH73), B27 supplement (Invitrogen, 12587–010), N2 supplement (Invitrogen, 17502–048), GlutaMAX (Invitrogen, 35050061), penicillin/streptomycin (Thermo Fisher Scientific, 15140122), 0.2% BSA (Sigma-Aldrich, B2064), 10 μg/mL 1-thioglycerol (MTG, Sigma-Aldrich, M6145), 50 μg/mL ascorbic acid (Sigma-Aldrich, A4544), 50 nM dexamethasone (Sigma-Aldrich, D4902), 0.1 mM 8-Bromo-cAMP (Sigma-Aldrich, B7880), and 0.1 mM 3-isobutyl-1-methylxanthine (IBMX) (Sigma-Aldrich, I5879) with 10 μM TGF-β inhibitor (A8301, Stemcell, 72024), and 10%FBS was added in the first 14 days to form spheres; 10 μM Y27632 was added in the first 2 days, and culture medium was replenished every other day. CHIR99021 was retracted from the culture medium from day 12, and 50 ng/mL human recombinant IFN-γ (Novoprotein, C014) or PBS was added from day 15 for 4 days. At day 18, the recombinant IFN-γ was reduced to 25 ng/mL, and organoids were collected at day 21 for analysis. For inhibitor treatment, cells were pretreated with indicated inhibitors from day 12. Forty-eight hours later, fresh medium containing IFN-γ and indicated inhibitors was replenished. The following molecules were used: 10 μM FAK inhibitor PF-573228 and 10 μM Src inhibitor dasatinib. Human MRC5 cells were cultured in medium containing DMEM-F12, 10%FBS, GlutaMAX, and 1% penicillin/streptomycin. MRC5 cells with passage of less than 5 were used. MRC5 cells with higher passages showed reduced ability to support human organoid growth.

### Human lung tissue.

Human lung tissues were obtained from the Cardiothoracic Surgery Department, Children’s Hospital of Fudan University, and the Department of Respiratory Medicine, Shanghai Jiao Tong University Affiliated Sixth People’s Hospital. For the complete list of human lung tissues, refer to Supplemental Tables 1 and 2.

### Quantitative reverse-transcription PCR.

For quantitative reverse-transcription PCR (qRT-PCR), the left lung tissues were collected and stored in Trizol (Vazyme, REC03B-100) prior to RNA isolation. cDNA was then synthesized using the HiScript III RT SuperMix for qPCR Kit (Vazyme, R323-01). ChamQ Universal SYBR qPCR Master Mix standard program was run following the manufacturer’s protocol (Vazyme, Q711-03) on a Roche LC480-384 instrument. Gene expression was calculated relative to tubulin within that sample and expressed as fold change over the average expression. All primers are listed in Supplemental Table 3.

### Statistics.

As indicated in the figure legends, 2-tailed *t* test, 2-tailed Mann-Whitney *U* test, or multiple *t* test was used for 2 groups. Levene’s test was used to check the equality of variances across groups, and then 1-way ANOVA or Brown-Forsythe and Welch’s ANOVA followed by the original FDR method of the Benjamini and Hochberg multiple-comparison test were used for more than 2 groups. A *P* value of less than 0.05 was considered significant. Data are represented as mean ± SEM. Statistical analysis was performed using Prism, version 10.2.0 (GraphPad Inc.).

### Study approval.

All animal experiments were conducted under IACUC-approved protocols at the Center for Excellence in Molecular Cell Science and the CAS Key Laboratory of Molecular Virology and Immunology, Shanghai Institute of Biochemistry and Cell Biology, Chinese Academy of Science. Human lung tissues were collected under the surgical criteria of the Cardiothoracic Surgery Department, Children’s Hospital of Fudan University, and the department of Respiratory Medicine, Shanghai Jiao Tong University Affiliated Sixth People’s Hospital. Human research was approved by the institutional review boards of Fudan University and the Department of Respiratory Medicine, Shanghai Jiao Tong University Affiliated Sixth People’s Hospital. All participants provided written, informed consent.

### Data availability.

The scRNA-Seq data generated in this study have been deposited in the NCBI’s Gene Expression Omnibus database (GEO GSE234082). Previously published scRNA-Seq data that are reanalyzed here are available at GSE171524. All analyses associated with this study used preexisting R packages. No custom code was generated. Values for all data points in graphs are reported in the Supporting Data Values file.

## Author contributions

XL and PS conceived and designed the experimental approach. XL, WC, HW, and PS performed experiments. XL, GY, JZ, and PS analyzed the data. WC provided human lung samples. ZL and TR provided COVID-19 samples. BL provided resources for IAV infection experiments. GY, YX, and BL reviewed and edited the manuscript. XL and PS wrote the manuscript with input from other authors.

## Supplementary Material

Supplemental data

Unedited blot and gel images

Supporting data values

## Version 1 10/01/2024

Electronic publication
