# Supplementary material for: Viral infection induces inflammatory signals that coordinate YAP regulation of dysplastic cells in lung alveoli
Source: J Clin Invest. 2024 Oct 1;134(19):e176828. doi: 10.1172/JCI176828 (PMC11444164; doi:10.1172/JCI176828)
Supplement: Supplemental data [file jci-134-176828-s064.pdf]

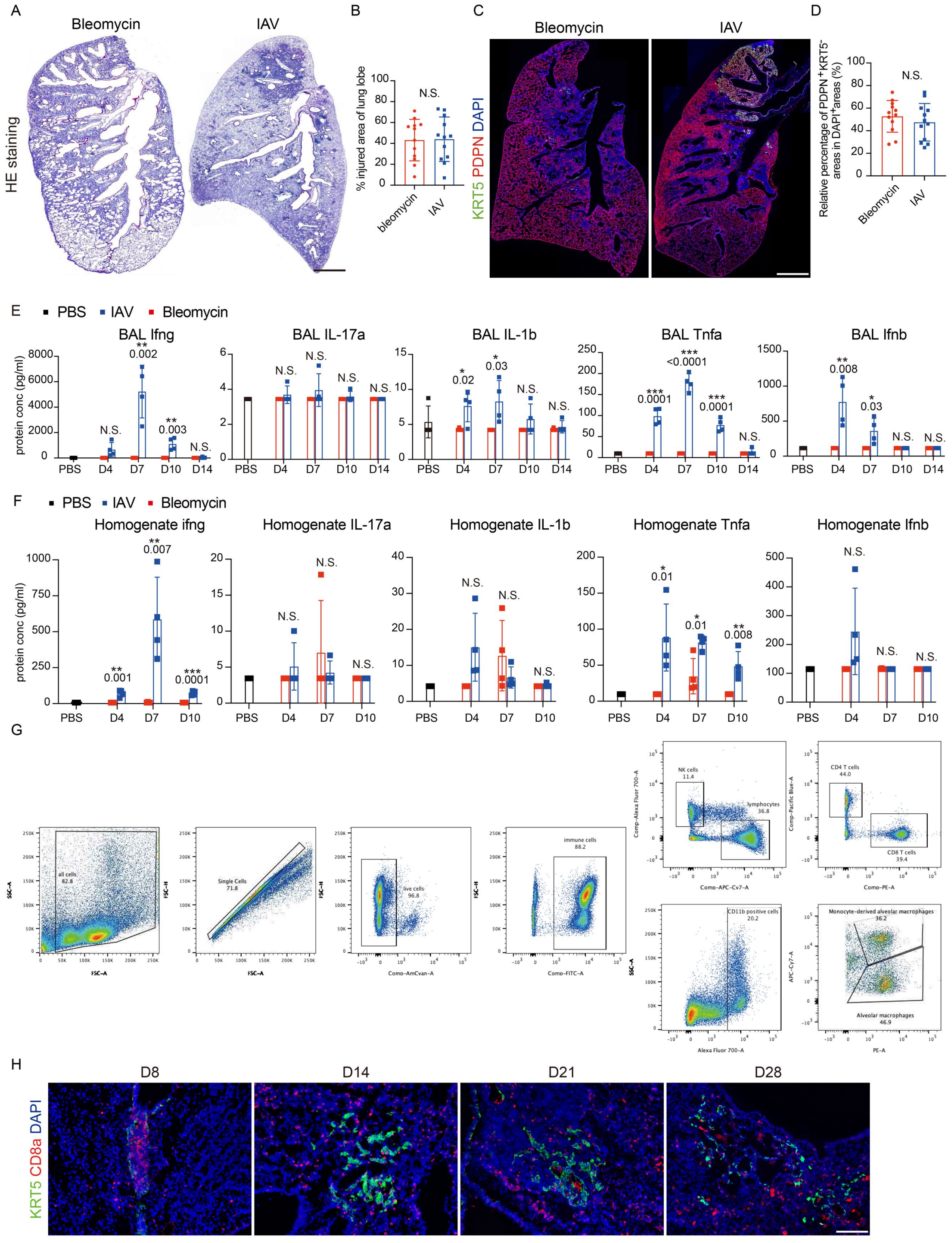

A
Bleomycin
IAV
HE staining
B
% injured area of lung lobe
N.S.
bleomycin
IAV
C
Bleomycin
IAV
KRT5 PDPN DAPI
D
Relative percentage of PDPN+KRT5- areas in DAPI+areas (%)
N.S.
Bleomycin
IAV
E
PBS IAV Bleomycin
BAL Ifng
BAL IL-17a
BAL IL-1b
BAL Tnfa
BAL Ifnb
protein conc (pg/ml)
PBS D4 D7 D10 D14
F
PBS IAV Bleomycin
Homogenate ifng
Homogenate IL-17a
Homogenate IL-1b
Homogenate Tnfa
Homogenate Ifnb
protein conc (pg/ml)
PBS D4 D7 D10
G
all cells 82.8
Single Cells 71.8
live cells 96.8
immune cells 88.2
NK cells 11.4
lymphocytes 36.8
CD4 T cells 44.0
CD8 T cells 39.4
CD11b positive cells 20.2
Monocyte-derived alveolar macrophages 36.2
Alveolar macrophages 46.9
H
D8
D14
D21
D28
KRT5 CD8a DAPI

Supplemental Figure 1. $CD8^+$ T cells persist in the $KRT5^+$ alveolar area after IAV infection. (**A**-**B**) Hematoxylin and eosin (H&E) staining and quantification of remodeling alveolar areas in IAV and bleomycin model (n = 12 mice per group). Scale bars: 500 μm. (**C**-**D**) Immunofluorescence analysis and quantification of percentages of $PDPN^+$ $KRT5^-$ areas in whole left lung areas ($DAPI^+$) in IAV and bleomycin model (n = 12 mice per group). Scale bars: 500 μm. (**E**) Inflammatory cytokines in BAL from IAV and bleomycin mice lung (n = 4 mice per group). (**F**) Inflammatory cytokines in IAV and bleomycin injured lung homogenate (n = 4 mice per group). (**G**) Representative plots for analyzing total lymphocytes, NK cells, $CD4^+$ T cells, $CD8^+$ T cells, alveolar macrophages, and monocyte-derived inflammatory macrophages. (**H**) Immunofluorescence images of $CD8^+$ T cells with $KRT5^+$ cells at indicated time points after IAV challenge. Data are representative of sections from 3 mice at each time point. Scale bars: 50 μm. * for $P < 0.05$; ** for $P < 0.01$; *** for $P < 0.001$. Error bars represent means ± SEM. Two-tailed Student's t test for **B** and **D**; Multiple t test for **E** and **F**.

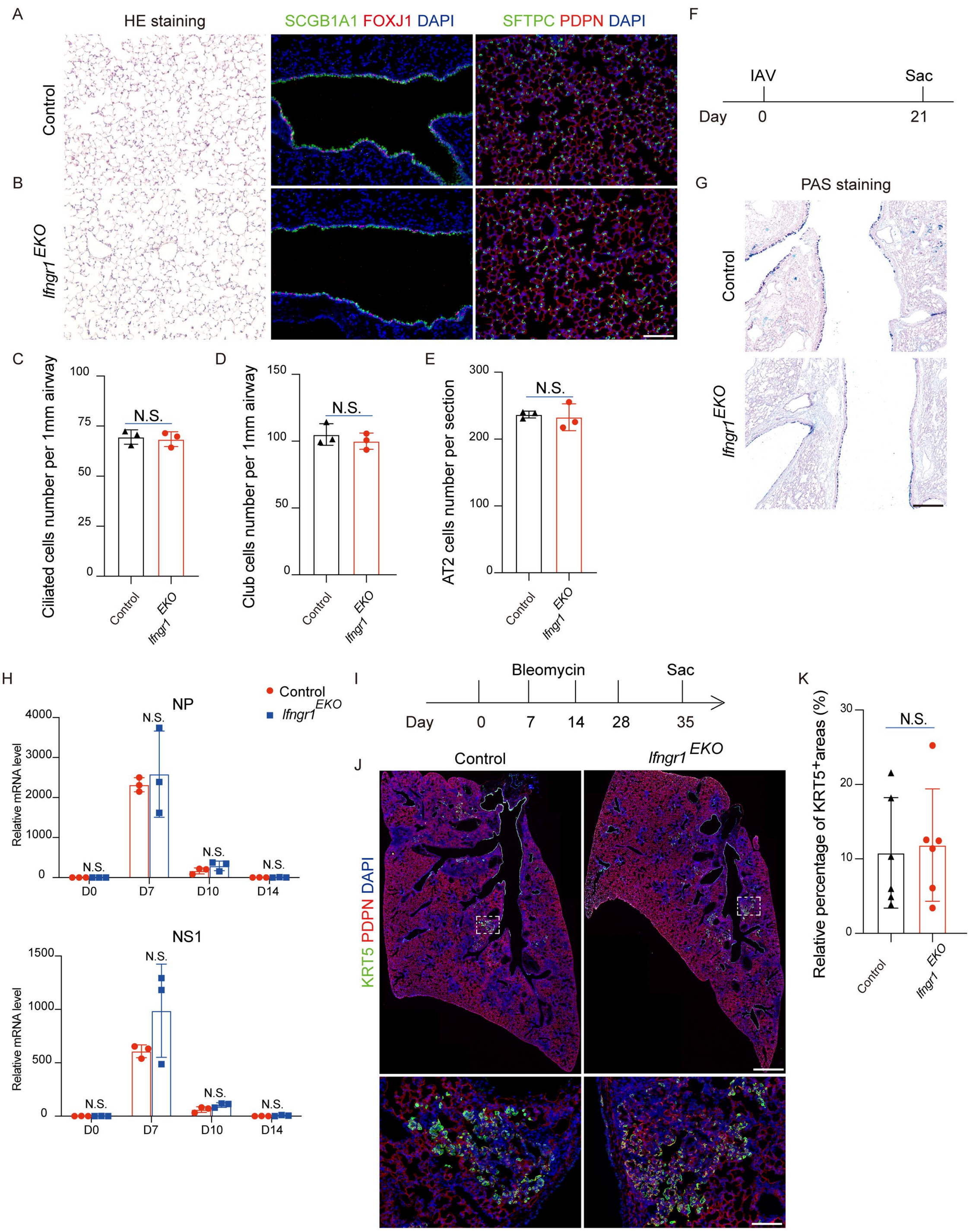

A
B
C
D
E
F
G
H
I
J
K
HE staining
SCGB1A1 FOXJ1 DAPI
SFTPC PDPN DAPI
Control
Ifngr1 EKO
Ciliated cells number per 1mm airway
Club cells number per 1mm airway
AT2 cells number per section
N.S.
IAV
Sac
Day 0 21
PAS staining
NP
NS1
Relative mRNA level
Control
Ifngr1 EKO
D0 D7 D10 D14
Bleomycin
Day 0 7 14 28 35
KRT5 PDPN DAPI
Relative percentage of KRT5+areas (%)

Supplemental Figure 2. The role of interferon signaling in homeostasis and in regulated dysplastic alveolar remodeling following IAV or bleomycin injury. (**A-E**) *Ifngr1*^{EKO} mutant mice showed comparable number of SCGB1A1^{+} club cells, FOXJ1^{+} ciliated cells, SFTPC^{+} AT2 cells, and PDPN^{+} AT1 cells as control mice (n = 3 mice per group). Scale bars: 50 μm. (**F**) Illustration of influenza A virus (IAV; PR8 strain) infection model. (**G**) PAS staining showing goblet cells hyperplasia in the airway in both control and *Ifngr1*^{EKO} mice after IAV challenge. Data are representative of sections from 3 mice respectively. Scale bars: 50 μm. (**H**) Expression of influenza specific genes, nucleoprotein (NP) and Non-Structural Protein 1 (NS1), as assayed by qRT-PCR in control and *Ifngr1*^{EKO} mice after IAV challenge at indicated time points (n = 3 mice per group). (**I**) Illustration of repetitive bleomycin challenge induced lung injury model. (**J**) Immunofluorescence images of dysplastic KRT5^{+} cells and PDPN^{+} AT1 cells in control, and *Ifngr1*^{EKO} mice lungs after repetitive bleomycin treatment. Scale bars: 500 μm (top row) and 50 μm (bottom row). (**K**) Quantification of percentages of KRT5^{+} alveolar area in total damaged lung area (PDPN^{-} and KRT5^{+}) in control and *Ifngr1*^{EKO} mice after bleomycin treatment (n = 6 mice per group). * for $P < 0.05$; ** for $P < 0.01$; *** for $P < 0.001$. Error bars represent means ± SEM.Two-tailed Student's t test for **C**-**E**; Multiple t test for **H**; two-tailed Mann–Whitney U test for **K**.

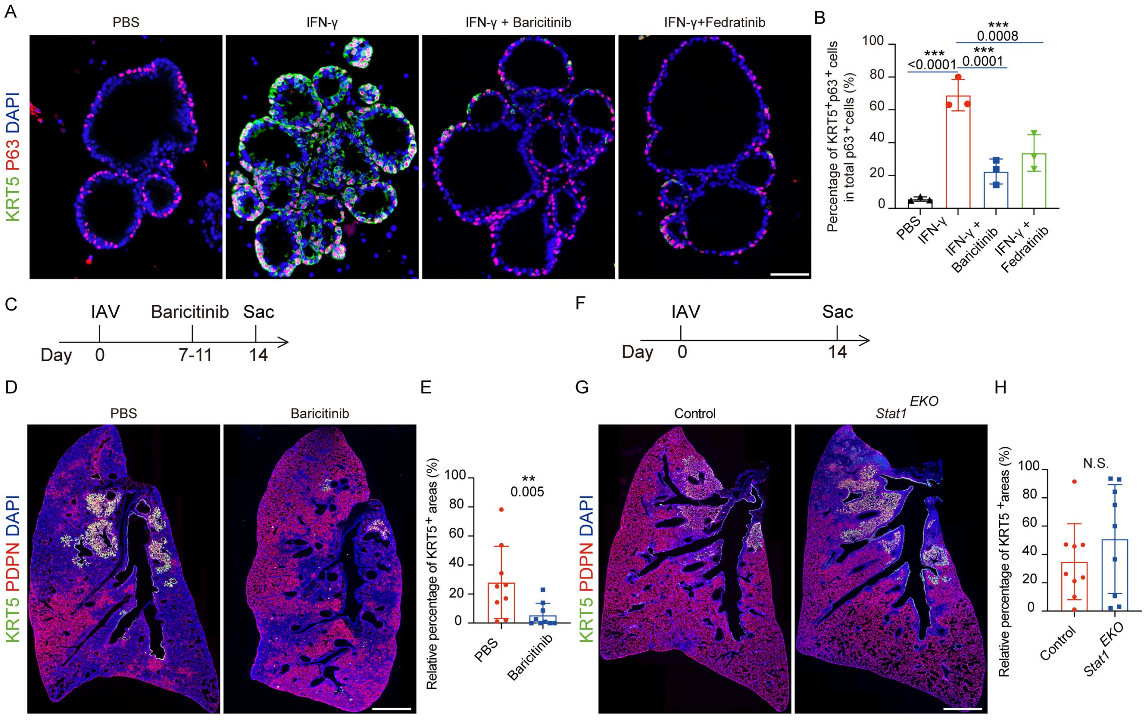
A
PBS
IFN-γ
IFN-γ + Baricitinib
IFN-γ+Fedratinib
KRT5 P63 DAPI
B
Percentage of KRT5+p63+ cells in total p63+ cells (%)
***
<0.0001
***
0.0001
***
0.0008
PBS
IFN-γ
IFN-γ + Baricitinib
IFN-γ + Fedratinib
C
IAV
Baricitinib
Sac
Day
0
7-11
14
D
PBS
Baricitinib
KRT5 PDPN DAPI
E
Relative percentage of KRT5+ areas (%)
**
0.005
PBS
Baricitinib
F
IAV
Sac
Day
0
14
G
Control
Stat1 EKO
KRT5 PDPN DAPI
H
Relative percentage of KRT5 +areas (%)
N.S.
Control
Stat1 EKO

Supplemental Figure 3. IFN-γ regulates lung dysplastic remodeling in a *Stat1* independent manner. (**A**-**B**) Immunofluorescence staining and quantification of percentages of $KRT5^{+}$ $p63^{+}$ cells in total $p63^{+}$ cells of PBS, IFN-γ, IFN-γ and Baricitinib, or IFN-γ and Fedratinib treated organoids (n = 3 technical replicates, experiment repeated twice). Scale bars: 25 μm. (**C**-**E**) Experiment design and quantification of percentages of $KRT5^{+}$ dysplastic cell areas in damage alveolar areas ($PDPN^{-}$ and $KRT5^{+}$) in control and Baricitinib mice at 14 dpi (n = 9 mice per group). Scale bars: 500 μm. (**F**-**H**) Experiment design and quantification of percentages of $KRT5^{+}$ dysplastic cell areas in damage alveolar areas ($PDPN^{-}$ and $KRT5^{+}$) in control and *Stat1*^{EKO} mutant mice at 14 dpi (n = 9 mice per group). Scale bars: 500 μm. * for $P < 0.05$; ** for $P < 0.01$; *** for $P < 0.001$. Error bars represent means ± SEM. One-way ANOVA for **B**; two-tailed Mann–Whitney U test for **E** and **H**.

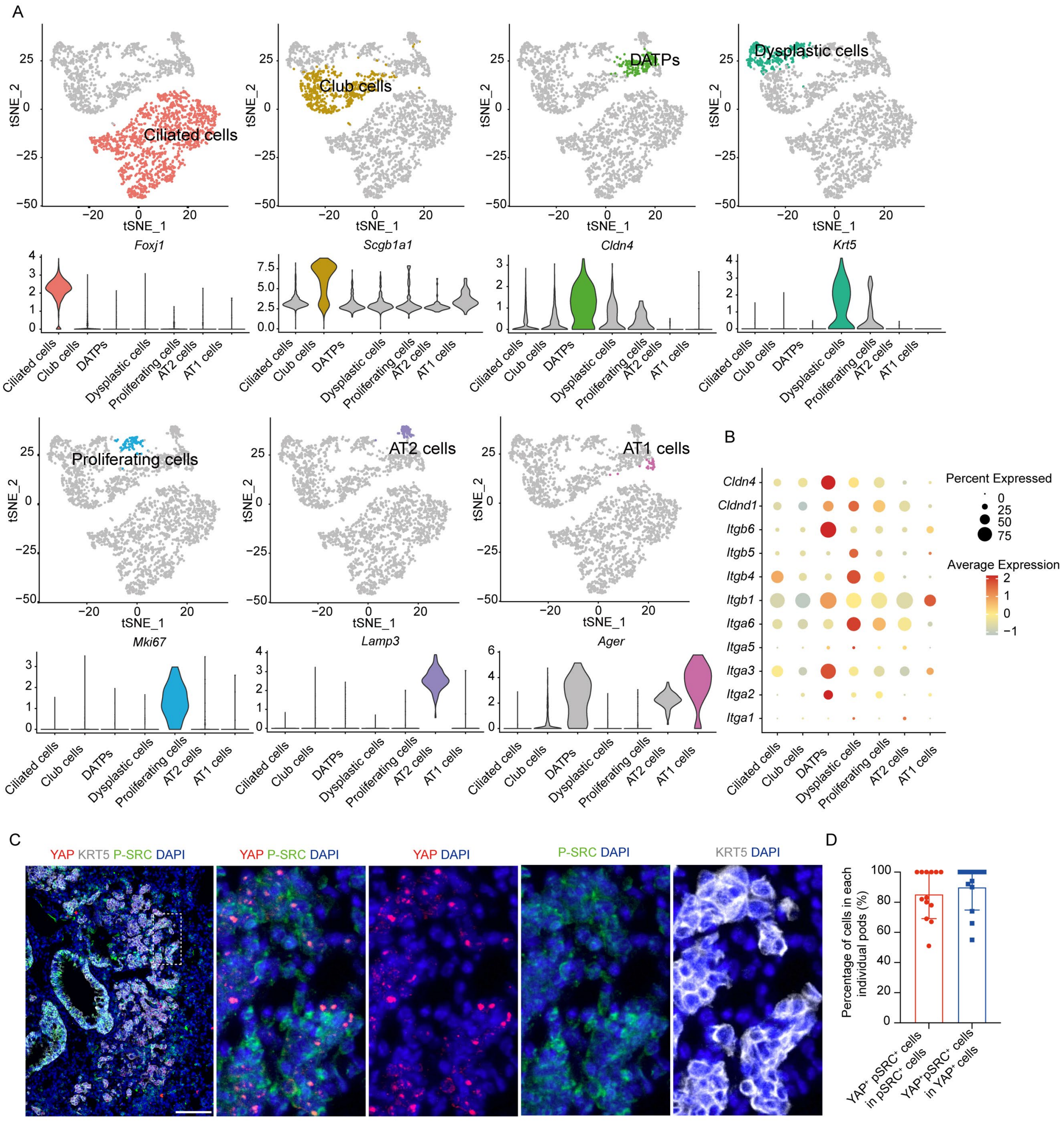
A
Ciliated cells
Club cells
DATPs
Dysplastic cells
Proliferating cells
AT2 cells
AT1 cells
tSNE_1
tSNE_2
Foxj1
Scgb1a1
Cldn4
Krt5
Mki67
Lamp3
Ager
B
Cldn4
Cldnd1
Itgb6
Itgb5
Itgb4
Itgb1
Itga6
Itga5
Itga3
Itga2
Itga1
Percent Expressed
Average Expression
C
YAP KRT5 P-SRC DAPI
YAP P-SRC DAPI
YAP DAPI
P-SRC DAPI
KRT5 DAPI
D
Percentage of cells in each individual pods (%)
YAP⁺ pSRC⁺ cells in pSRC⁺ cells
YAP⁺pSRC⁺ cells in YAP⁺ cells

Supplemental Figure 4. Single-cell analysis of lung epithelial cells from IAV infected lungs. (**A**) t-SNE showing the expression of marker genes in each cell cluster. (**B**) Dot plot showing the expression of integrin and focal adhesion related genes in all epithelial cells. (**C**-**D**) Immunofluorescence staining and quantification of percentages of YAP^+pSRC^+ cells in $pSRC^+$ cells or YAP^+pSRC^+ cells in YAP^+ cells in each individual pods at 12 dpi (n = 13 pods from 5 mice). Scale bars: 50 μm. * for $P < 0.05$; ** for $P < 0.01$; *** for $P < 0.001$. Error bars represent means ± SEM. Two-tailed Mann–Whitney U test for **E**.

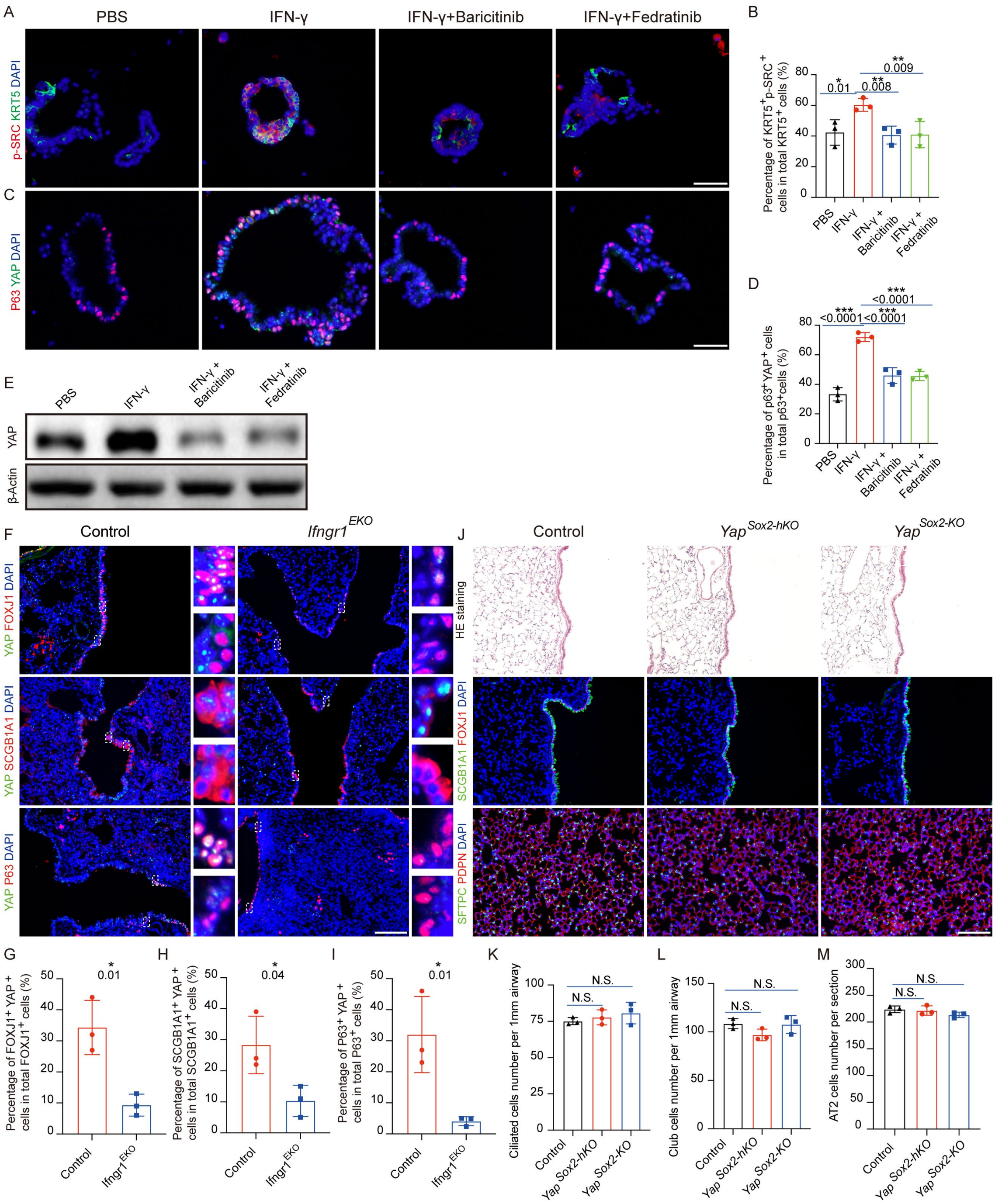

A
PBS
IFN-γ
IFN-γ+Baricitinib
IFN-γ+Fedratinib
p-SRC KRT5 DAPI
B
Percentage of KRT5+p-SRC+ cells in total KRT5+ cells (%)
C
P63 YAP DAPI
D
Percentage of p63+YAP+ cells in total p63+cells (%)
E
YAP
β-Actin
F
Control
Ifngr1 EKO
YAP FOXJ1 DAPI
YAP SCGB1A1 DAPI
YAP P63 DAPI
J
Control
Yap Sox2-hKO
Yap Sox2-KO
HE staining
SCGB1A1 FOXJ1 DAPI
SFTPC PDPN DAPI
G
Percentage of FOXJ1+ YAP+ cells in total FOXJ1+ cells (%)
H
Percentage of SCGB1A1+ YAP+ cells in total SCGB1A1+ cells (%)
I
Percentage of P63+ YAP+ cells in total P63+ cells (%)
K
Ciliated cells number per 1mm airway
L
Club cells number per 1mm airway
M
AT2 cells number per section
N.S.

Supplemental Figure 5. IFN-γ promote p-SRC and YAP activation. (**A**-**B**) Immunofluorescence staining and quantification of $KRT5^+$ $pSRC^+$ cells in $KRT5^+$ cells treated with PBS, IFN-γ, IFN-γ and JAK inhibitor Baricitinib or Fedratinib (n = 3 technical replicates, experiment repeated twice). Scale bars: 25 μm. (**C**-**D**) Immunofluorescence images and quantification of the number of YAP^+ $p63^+$ cells in cultured mouse intrapulmonary $p63^+$ cells treated with PBS, IFN-γ, IFN-γ and JAK inhibitor Baricitinib or Fedratinib (n = 3 technical replicates, experiment repeated twice). Scale bars: 25 μm. (**E**) Western blot analysis of YAP protein in cultured intrapulmonary $p63^+$ cells treated with PBS, IFN-γ, IFN-γ and Baricitinib, IFN-γ and Fedratinib. (**F**-**I**) Immunofluorescence staining and quantification of cells expressing nuclear YAP in control and *Ifngr1*^{EKO} mice at 14 dpi (n = 3 mice per group). Scale bars: 50 μm. (**J**) H&E staining or immunofluorescence images of $SCGB1A1^+$ club cells, $FOXJ1^+$ ciliated cells, $SFTPC^+$ AT2 cells, and $PDPN^+$ AT1 cells in control and mutant mice at homeostasis. Scale bars: 50 μm. (**K**-**M**) Quantification of number of $SCGB1A1^+$ club cells, $FOXJ1^+$ ciliated cells, and $SFTPC^+$ AT2 cells in control and mutant mice at homeostasis (n = 3 mice per group). * for $P < 0.05$; ** for $P < 0.01$; *** for $P < 0.001$. Error bars represent means ± SEM. One-way ANOVA for **B** and **D**, **K**-**M**; two-tailed Student's t test for **G**-**I**.

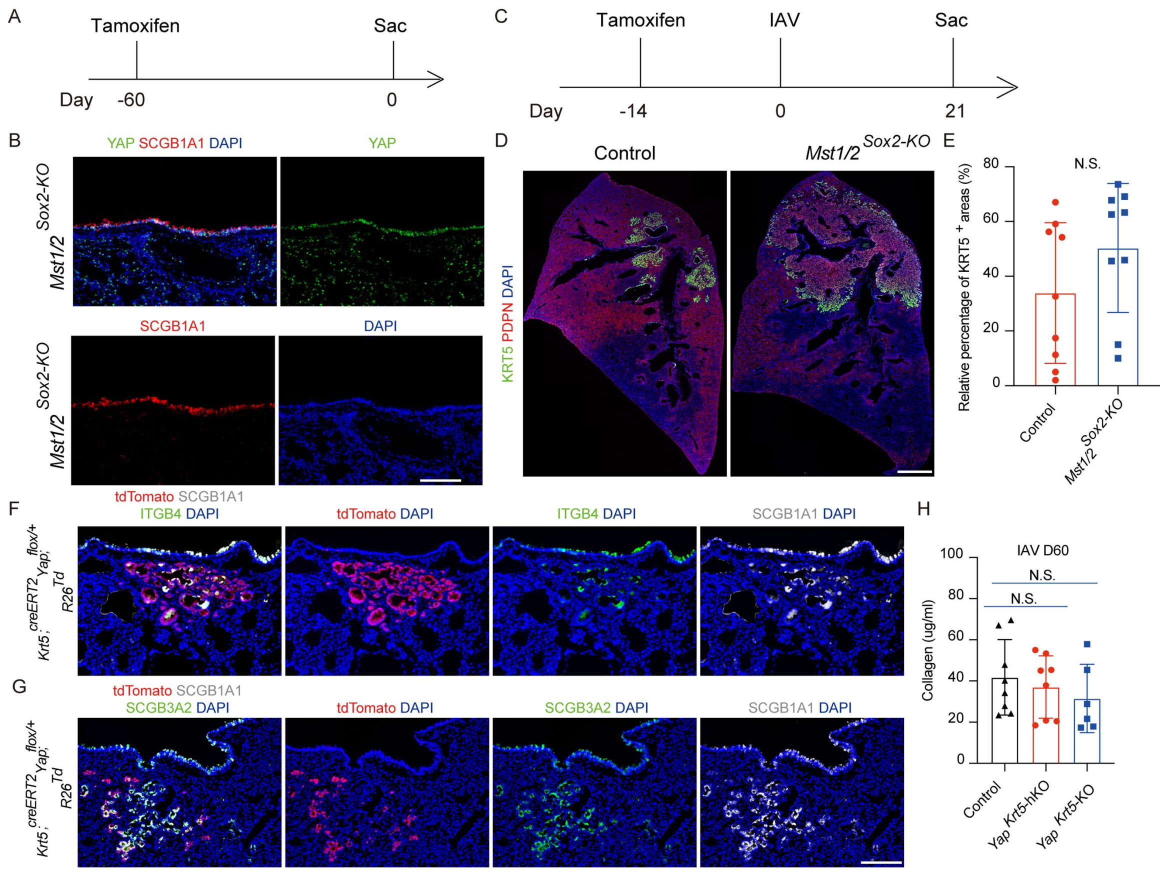
A
Tamoxifen
Sac
Day -60 0
C
Tamoxifen
IAV
Sac
Day -14 0 21
B
YAP SCGB1A1 DAPI
YAP
SCGB1A1
DAPI
Mst1/2 Sox2-KO
D
Control
Mst1/2 Sox2-KO
KRT5 PDPN DAPI
E
N.S.
Relative percentage of KRT5 + areas (%)
80
60
40
20
0
Control
Mst1/2 Sox2-KO
F
tdTomato SCGB1A1 ITGB4 DAPI
tdTomato DAPI
ITGB4 DAPI
SCGB1A1 DAPI
Krt5 creERT2; Yap flox/+; R26 Td
G
tdTomato SCGB1A1 SCGB3A2 DAPI
tdTomato DAPI
SCGB3A2 DAPI
SCGB1A1 DAPI
Krt5 creERT2; Yap flox/+; R26 Td
H
IAV D60
N.S.
N.S.
Collagen (ug/ml)
100
80
60
40
20
0
Control
Yap Krt5-hKO
Yap Krt5-KO

Supplemental Figure 6. Further validation of YAP function in regulating dysplastic cells. (**A**-**B**) Experiment design and immunofluorescence images of YAP and SCGB1A1 in *Mst1*/*Mst2*$^{Sox2\text{-KO}}$ mutant mice at homeostasis. Data are representative of sections from 3 mice. Scale bars: 50 μm. (**C**-**D**) Experiment design and immunofluorescence images of dysplastic KRT5^{+} cells and PDPN^{+} AT1 in control and *Mst1*/*Mst2*$^{Sox2\text{-KO}}$ mutant mice lungs after IAV challenge. Scale bars: 500 μm. (**E**) Quantification of the percentages of KRT5^{+} alveolar area in total damaged lung area (PDPN^{-} and KRT5^{+}) in control and *Mst1*/*Mst2*$^{Sox2\text{-KO}}$ mutant mice lungs after IAV challenge (n = 9 mice per group). (**F**-**G**) Representative IHC showing ITGB4 and SCGB3A2 expressing in lineage-traced SCGB1A1^{+} cells in $Krt5^{creERT2/+}$;$Yap^{flox/+}$;$R26^{Td}$ mice at 35 dpi. Data are representative of sections from 3 mice respectively. Scale bars: 50 μm. (**H**) Quantitative analysis of collagen content in lung homogenates from control, $Yap^{Krt5\text{-hKO}}$, and $Yap^{Krt5\text{-KO}}$ mice at 60 dpi (Tamoxifen at day 21 post infection) (n ≥ 6 mice per group). * for $P < 0.05$; ** for $P < 0.01$; *** for $P < 0.001$. Error bars represent means ± SEM. Two-tailed Mann–Whitney U test for **E**; One-way ANOVA for **H**.

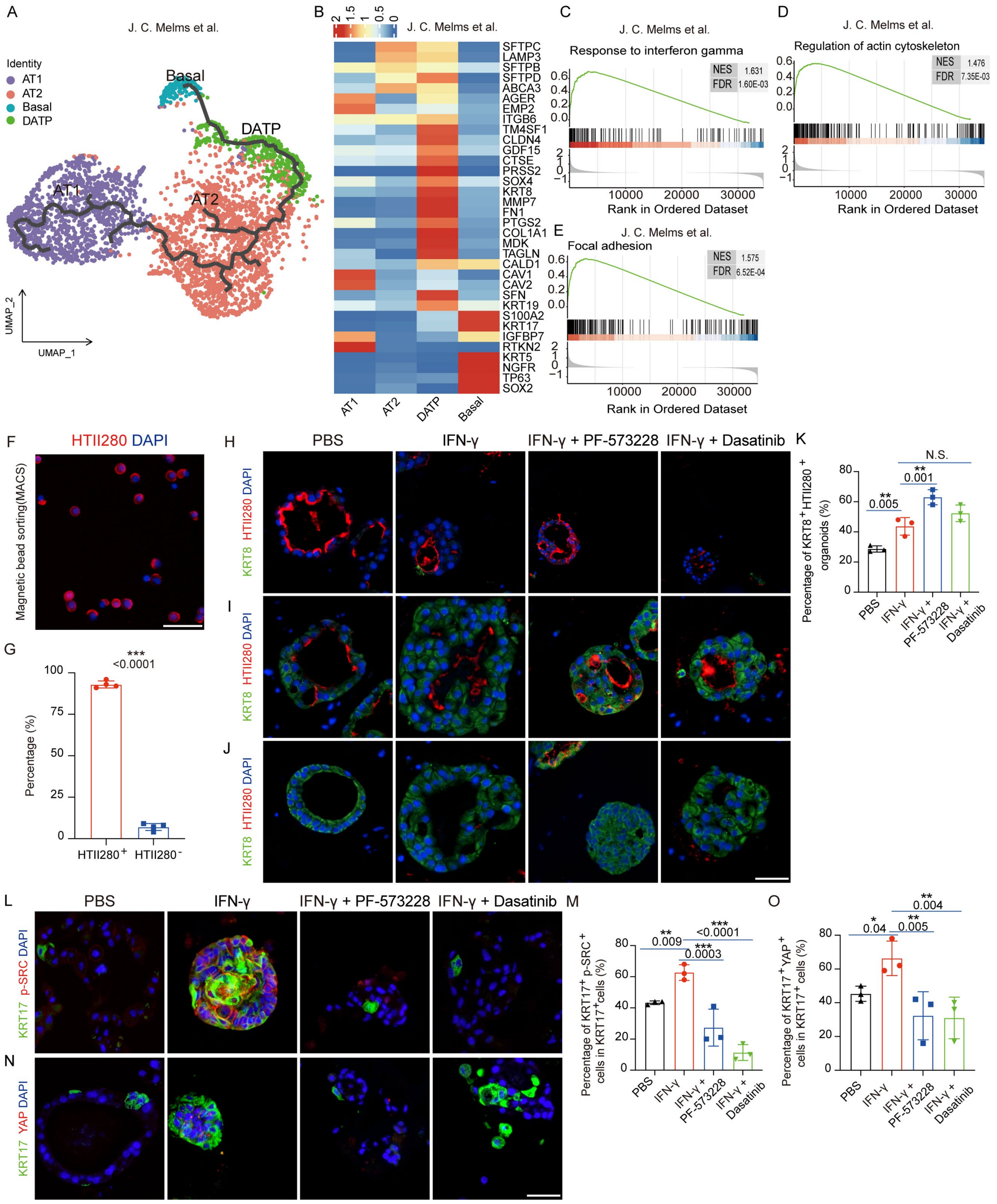
A
J. C. Melms et al.
Identity
AT1
AT2
Basal
DATP
Basal
DATP
AT1
AT2
UMAP_2
UMAP_1
B
2
1.5
1
0.5
0
J. C. Melms et al.
SFTPC
LAMP3
SFTPB
SFTPD
ABCA3
AGER
EMP2
ITGB6
TM4SF1
CLDN4
GDF15
CTSE
PRSS2
SOX4
KRT8
MMP7
FN1
PTGS2
COL1A1
MDK
TAGLN
CALD1
CAV1
CAV2
SFN
KRT19
S100A2
KRT17
IGFBP7
RTKN2
KRT5
NGFR
TP63
SOX2
AT1
AT2
DATP
Basal
C
J. C. Melms et al.
Response to interferon gamma
NES 1.631
FDR 1.60E-03
Rank in Ordered Dataset
D
J. C. Melms et al.
Regulation of actin cytoskeleton
NES 1.476
FDR 7.35E-03
Rank in Ordered Dataset
E
J. C. Melms et al.
Focal adhesion
NES 1.575
FDR 6.52E-04
Rank in Ordered Dataset
F
HTII280 DAPI
Magnetic bead sorting(MACS)
G
***
<0.0001
Percentage (%)
HTII280+
HTII280-
H
PBS
IFN-γ
IFN-γ + PF-573228
IFN-γ + Dasatinib
KRT8 HTII280 DAPI
I
J
K
N.S.
**
0.001
**
0.005
Percentage of KRT8+HTII280+ organoids (%)
PBS
IFN-γ
IFN-γ + PF-573228
IFN-γ + Dasatinib
L
PBS
IFN-γ
IFN-γ + PF-573228
IFN-γ + Dasatinib
KRT17 p-SRC DAPI
N
KRT17 YAP DAPI
M
**
0.009
***
<0.0001
***
0.0003
Percentage of KRT17+ p-SRC+ cells in KRT17+cells (%)
O
**
0.004
*
0.04
**
0.005
Percentage of KRT17+YAP+ cells in KRT17+cells (%)

Supplemental Figure 7. SC-RNA seq analysis for Covid19 lung epithelial and human organoid culture. (**A**) Single-cell RNA-Seq t-SNE clustering of lung epithelial cells from Covid-19 patient. (**B**) Heatmap showing marker genes expression in each cell cluster. (**C**-**E**) Gene Set Enrichment Analysis (GSEA) revealed that in Covid-19 lungs, response to IFN-γ, regulation of actin cytoskeleton, and focal adhesion pathway were highly activated in KRT8$^+$ dysplastic cells compared to in AT2 cells. (**F**) Magnetic bead sorting (MACS) HTII 280$^+$ human AT2 cells. Scale bars: 25 μm. (**G**) Purity of sorted HTII-280$^+$ cells (n = 4 biological replicates). (**H**-**J**) Representative images of HTII-280$^+$ KRT8$^-$, HTII-280$^+$ KRT8$^+$, and HTII-280$^-$ KRT8$^+$ organoids from each group. Scale bars: 25 μm. (**K**) Quantification of KRT8$^+$ HTII-280$^+$ organoids in total human AT2 organoids treated with PBS, IFN-γ, IFN-γ and SRC inhibitor (Dasatinib) or IFN-γ and FAK inhibitor (PF-573228) (n = 3 technical replicates, experiment repeated twice). (**L**-**O**) Immunofluorescence staining and quantification of percentages of p-SRC$^+$KRT17$^+$ cells and YAP$^+$KRT17$^+$ cells in KRT17$^+$ cells from human AT2 organoid treated with PBS, IFN-γ, IFN-γ and SRC inhibitor (Dasatinib) or IFN-γ and FAK inhibitor (PF-573228) (n = 3 technical replicates, experiment repeated twice). Scale bars: 25 μm. * for $P < 0.05$; ** for $P < 0.01$; *** for $P < 0.001$. Error bars represent means ± SEM. Two-tailed Student's t test for **G;** One-way ANOVA for **K**, **M**, and **O**.

Supplemental Table 1.
Human lung Information

Donor (D)	Age	Sex	disease
D1	14	F	pneumothorax
D2	13	F	pneumothorax
D3	14	F	Pulmonary bullae
D4	10	F	Secondary Lung Tumors
D5	16	M	pneumothorax
D6	6	M	Mediastinal Tumor (Mass)
D7	13	M	pneumothorax

Supplemental Table 2.
Covid19 patient lung tissue Information

**Patient (P)**	**Age**	**Sex**	**disease**
P1	63	F	Covid19
P2	54	M	Covid19

Supplemental Table 3.

qPCR primers

Ifng	5’ - GAGGAACTGGCAAAAGGATGGT -3’<br>5’ - TTTCGCCTTGCTGTTGCTGA -3’
Ifnb	5’ - CCTGGAGCAGCTGAATGGAA -3’<br>5’ - CCACCCAGTGCTGGAGAAAT -3’
Il5	5’- AACTGTCCGTGGGGGTACT -3’<br>5’- CTCGCCACACTTCTCTTTTTGG -3’
Il22	5’- GTGAGAAGCTAACGTCCATCATT -3’<br>5’- CTGGTCTCATGGACAACTTGA -3’
Il1b	5’- TGCCACCTTTTGACAGTGATG -3’<br>5’- TGATGTGCTGCTGCGAGATT -3’
Il17a	5’- ACCCTGGACTCTCCACCGCAA -3’<br>5’- GGTGGTCCAGCTTTCCCTCCG -3’
Il13	5’-AAAGCAACTGTTTCGCCACG-3’<br>5’-CCTCTCCCCAGCAAAGTCTG-3’
Tnfa	5’- TAGCCCACGTCGTAGCAAAC -3’<br>5’- ACAAGGTACAACCCATCGGC -3’
Krt5	5’- GCAGACACACGTCTCTGACA -3’<br>5’- TGCAGCTCCTCATACTTGGT -3’
PR8-NP	5’- ACGGCTGGTCTGACTCACAT -3’<br>5’- TCCATTCCGGTGCGAACAAG -3’
PR8-NS1	5’- AGCAGATAGTGGAGCGGATT -3’<br>5’- GTACAGAGGCCATGGTCATT -3’
Tubb1	5’- CGGCCAGGTCATCACTATTGGCAAC -3’<br>5’- GCCACAGGATTCCATACCCAAGAAG -3’
