# Supplementary figures and images for: Viral infection induces inflammatory signals that coordinate YAP regulation of dysplastic cells in lung alveoli
Source: J Clin Invest. 2024 Oct 1;134(19):e176828. doi: 10.1172/JCI176828 (PMC11444164; doi:10.1172/JCI176828)

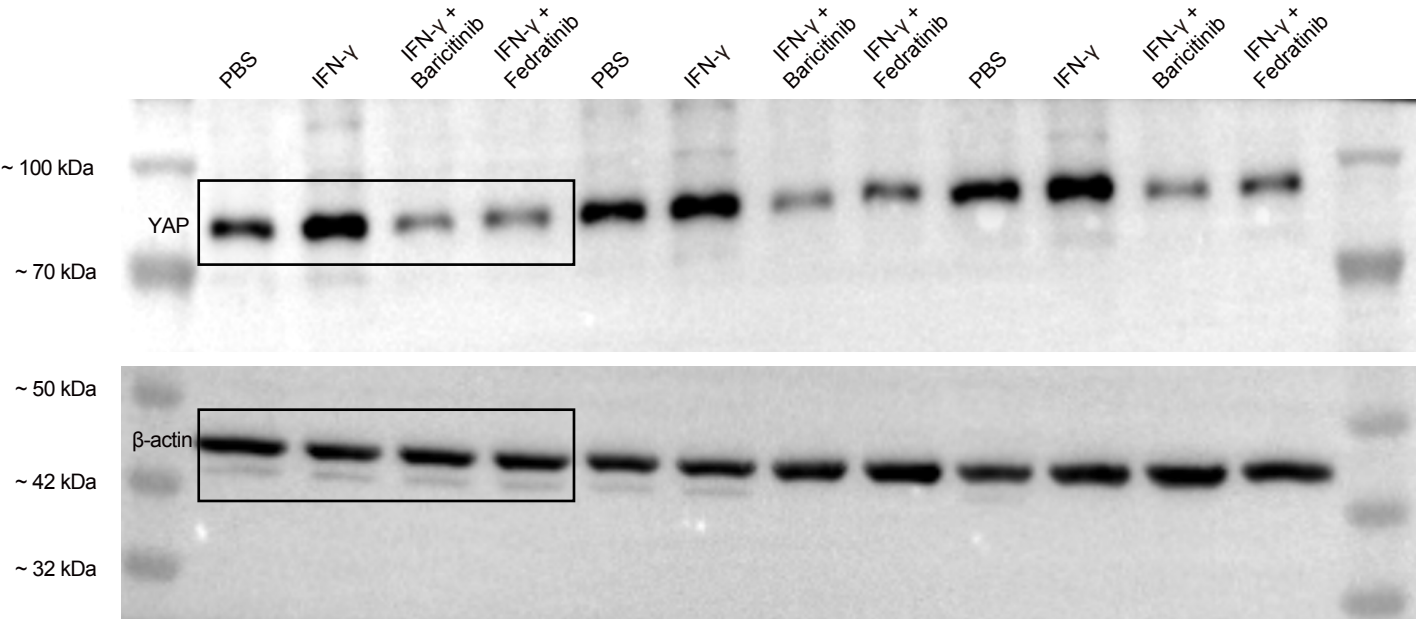

The lanes in the text box are shown in the Supplemental Figure 5E

Supplement: Unedited blot and gel images [file jci-134-176828-s065.pdf]
